# sPLINK: a hybrid federated tool as a robust alternative to meta-analysis in genome-wide association studies

**DOI:** 10.1186/s13059-021-02562-1

**Published:** 2022-01-24

**Authors:** Reza Nasirigerdeh, Reihaneh Torkzadehmahani, Julian Matschinske, Tobias Frisch, Markus List, Julian Späth, Stefan Weiss, Uwe Völker, Esa Pitkänen, Dominik Heider, Nina Kerstin Wenke, Georgios Kaissis, Daniel Rueckert, Tim Kacprowski, Jan Baumbach

**Affiliations:** 1 AI in Medicine and Healthcare, Technical University of Munich, Munich, Germany; 2 Klinikum rechts der Isar, Munich, Germany; 3 Chair of Computational Systems Biology, University of Hamburg, Hamburg, Germany; 4 Department of Mathematics and Computer Science, University of Southern Denmark, Odense, Denmark; 5 Chair of Experimental Bioinformatics, TUM School of Life Sciences, Technical University of Munich, Munich, Germany; 6 Department of Functional Genomics, University Medicine Greifswald, Greifswald, Germany; 7 Institute for Molecular Medicine Finland (FIMM), Helsinki Institute of Life Science (HiLIFE), University of Helsinki, Helsinki, Finland; 8 Applied Tumor Genomics Research Program, Research Programs Unit, Faculty of Medicine, University of Helsinki, Helsinki, Finland; 9 Department of Mathematics and Computer Science, University of Marburg, Marburg, Germany; 10 Division Data Science in Biomedicine, Peter L. Reichertz Institute for Medical Informatics of TU Braunschweig and Hannover Medical School, Brunswick, Germany; 11 Braunschweig Integrated Centre of Systems Biology (BRICS), Brunswick, Germany; 12 Biomedical Image Analysis Group, Imperial College London, London, UK; 13 OpenMined, Oxford, UK

**Keywords:** sPLINK, PLINK, Federated learning, Genome-wide association studies, GWAS, Meta-analysis, Privacy

## Abstract

Meta-analysis has been established as an effective approach to combining summary statistics of several genome-wide association studies (GWAS). However, the accuracy of meta-analysis can be attenuated in the presence of cross-study heterogeneity. We present *sPLINK*, a hybrid federated and user-friendly tool, which performs privacy-aware GWAS on distributed datasets while preserving the accuracy of the results. *sPLINK* is robust against heterogeneous distributions of data across cohorts while meta-analysis considerably loses accuracy in such scenarios. *sPLINK* achieves practical runtime and acceptable network usage for chi-square and linear/logistic regression tests. *sPLINK* is available at https://exbio.wzw.tum.de/splink.

**Supplementary Information:**

The online version contains supplementary material available at (10.1186/s13059-021-02562-1).

## Background

Genome-wide association studies (GWAS) test millions of single nucleotide polymorphisms (SNPs) to identify possible associations between a specific SNP and disease [1]. They have led to considerable achievements over the past decade including better comprehension of the genetic structure of complex diseases and the discovery of SNPs playing a role in many traits or disorders [2, 3]. GWAS sample size is an important factor in detecting associations, and larger sample sizes lead to identifying more associations and more accurate genetic predictors [2, 4].

**Fig. 1:**
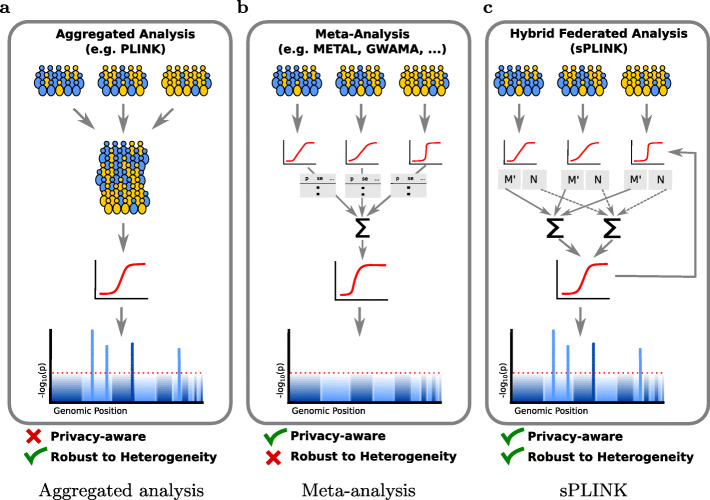
Comparison of *sPLINK* (**c**), aggregated analysis (**a**), and meta-analysis (**b**) approaches: Aggregated analysis requires cohorts to pool their private data for a joint analysis. The meta-analysis approaches aggregate the summary statistics from the cohorts to estimate the combined *p*-values. In *sPLINK*, the cohorts calculate the model parameters (*M*) from the local data and global model, generate noise (*N*), and make the parameters noisy (*M*^′^) in an iterative manner. The aggregated noise and noisy parameters are in turn aggregated to update the global model or build the final model. *sPLINK* combines the advantages of the aggregated analysis and meta-analysis, i.e. robustness against heterogeneous data and enhancing the privacy of cohorts’ data. Yellow/blue color indicates case/control samples

*PLINK* [5] is a widely used open source software tool for GWAS. The major limitation of *PLINK* is that it can only perform association tests on local data. If multiple cohorts want to conduct collaborative GWAS to take advantage of larger sample sizes, they can pool their data for a joint analysis (Fig. 1a); however, this is close to impossible due to privacy restrictions and data protection issues, especially concerning genetic and medical data. Hence, the field has established methods for meta-analysis of individual studies, where only the results and summary statistics of the individual analyses have to be exchanged [6] (Fig. 1b).

There are several software packages such as *METAL* [7], *GWAMA* [8], and *PLINK* [5] that implement different meta-analysis models including fixed or random effect models [9]. Although meta-analysis approaches are privacy-aware, i.e. the raw data is not shared with third parities, they suffer from two main constraints: first, they rely on detailed planning and agreement of cohorts on various study parameters such as meta-analysis model (e.g. fixed effect or random effect), meta-analysis tool (e.g., METAL or GWAMA), heterogeneity metric (e.g. Cochran’s *Q* or the *I*^2^ statistic), the covariates to be considered, etc [4]. Second and more importantly, the statistical power of meta-analysis can be adversely affected in the presence of cross-study heterogeneity, leading to inaccurate estimation of the joint results and yielding misleading conclusions [10, 11].

To address the aforementioned shortcomings, privacy-aware collaborative GWAS can be developed using homomorphic encryption (HE) [12], secure multi-party computation (SMPC) [13], and federated learning [14, 15]. In HE, the cohorts encrypt their private data and share it with a single server, which performs operations on the encrypted data from the cohorts to compute the association test results. In SMPC, there are several computing parties and the cohorts extract a separate secret share (anonymized chunk) [16] from the private data and send it to a computing party. The computing parties calculate intermediate results from the secret shares and exchange the intermediate results with each other. Each computing party computes the final results given all intermediate results. In federated learning, the cohorts extract model parameters (e.g. Hessian matrices) from the private data and share the parameters with a central server. The server aggregates the parameters from all cohorts to calculate the association test results.

Kamm et al. [17] and Cho et al. [18] proposed GWAS frameworks based on SMPC. The former developed simple association tests including Cochran–Armitage and chi-square (*χ*^2^) and the latter implemented only the Cochran–Armitage test for trend. Shi et al. [19] presented an SMPC-based logistic regression framework for GWAS. Constable et al. [20] implemented an SMPC-based framework for minor allele frequency and chi-square computation. These frameworks inherit the limitations of SMPC itself: They follow the paradigm of “move data to computation,” where they put the processing burden on a few computing parties. Consequently, they are computationally expensive [21] and are not scalable for large-scale GWAS. Moreover, they suffer from the colluding-parties problem [17] in which, if the parties send the secret shares of the cohorts to each other, the whole private data of the cohorts is exposed.

Lu et al. [22], Morshed et al. [23], and Kim et al. [24] developed chi-square, linear regression, and logistic regression tests using HE for GWAS, respectively. Sadat et al. [25] introduced the *SAFETY* framework based on HE and Intel Software Guard Extensions technology, which implements the linkage disequilibrium, Fisher’s exact test, Cochran-Armitage test for trend, and Hardy-Weinberg equilibrium statistical tests. Similar to SMPC-based methods, they are not computationally efficient because a single server carries out operations over encrypted data, causing considerable overhead [26]. Additionally, HE-based methods introduce accuracy loss in the association test results [23, 24]. This is because HE only supports addition and multiplication, and as a result, non-linear operations in regression tests should be approximated using those two operations.

To address the computational limitation of HE/SMPC-based methods, the association tests can be implemented in a federated fashion. Federated learning-based methods follow the paradigm of “move computation to data,” distributing the heavy computations among the cohorts while performing lightweight aggregation (simple operations such as addition and multiplication of the parameters) at the central server. Wang et al. [27] introduced EXPLORER for distributed logistic regression algorithm. EXPLORER is a model but not a tool for GWAS. Moreover, it does not provide a “guarantee for optimal global solution,” implying that its results can be different from the aggregated analysis in general. GLORE [28, 29] implemented a federated logistic regression test but the parameter values computed by each cohort are revealed to the server.

Several hybrid federated frameworks including *HyFed* [30] have been introduced to improve the privacy of federated learning by hiding the local parameters of a cohort from third parties. HyFed is a suitable framework for developing federated GWAS algorithms because it provides enhanced privacy while preserving the accuracy of the results. It also supports federated mode, where different components can run in separate physical machines and securely communicate with each other over the Internet.

**Fig. 2:**
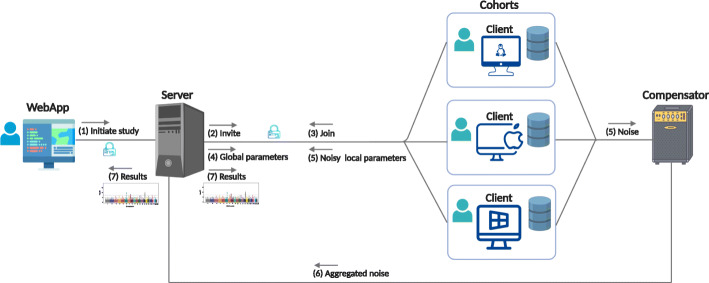
*Architecture of sPLINK*: (1) The coordinator creates a new project through the WebApp component and (2) invites a set of cohorts to join the project; (3) the cohorts join the project and select the dataset using the client component. The project is started automatically, when all cohorts joined. The computation of the test results is performed in a an iterative manner, where the clients (4) obtain the global parameters from the server, (5) compute the local parameters, mask them with noise, and share the noise and noisy local parameters with the compensator and server, respectively; (6) the compensator aggregates the noise values and sends the aggregated noise to the server; the server calculates the global parameters by aggregating the noisy local parameters and the negative of the aggregated noise; (7) after the computation is done, the cohorts and coordinator can access the results. All communications are performed in a secure channel over HTTPS protocol. The cohorts can use Linux distributions, Microsoft Windows, or MacOS to run the client component

In this paper, we present a hybrid federated tool called *sPLINK (safe PLINK)* based on the *HyFed* framework for privacy-aware GWAS. *sPLINK* consists of four main components (Fig. 2): *Web application (WebApp)* to configure the parameters (e.g. association test) of the new study; *client* to compute the local parameters, mask them with noise, and share the noise with *compensator* and noisy local parameters with *server*; *compensator* to aggregate the noise values of the clients and send the aggregated noise to the *server*; *server* to compute the global parameters by adding up the noisy local parameters and the negative of the aggregated noise. Notice that the utility of the global model is preserved because the aggregated noise from the compensator cancels out the accumulated noise from the noisy local parameters during the aggregation.

Unlike *PLINK*, *sPLINK* is applicable to distributed data in a privacy-aware fashion. In *sPLINK*, neither the private data of cohorts leaves the site nor the original values of the local parameters are revealed to the other parties (Fig. 1c). Contrary to the existing HE/SMPC-based methods, *sPLINK* is computationally efficient because heavy computations are distributed across the cohorts while simple aggregation is performed on the server and compensator. Compared to the current federated tools like GLORE, *sPLINK* not only provides enhanced privacy but also supports multiple association tests including logistic and linear regression [31], and chi-square [32] for GWAS.

The advantage of *sPLINK* over the meta-analysis approaches is twofold: usability and robustness against heterogeneity. *sPLINK* is easier to use for collaborative GWAS compared to meta-analysis. In *sPLINK*, a coordinator initiates a collaborative study and invites the cohorts. The only decision the cohorts make is whether or not to join the study. After accepting the invitation, the cohorts just select the dataset they want to employ in the study. More importantly, *sPLINK* is robust to data heterogeneity (phenotype and confounding factors). It gives the same results as aggregated analysis even if the phenotype distribution is imbalanced or if confounding factors are distributed heterogeneously across cohorts. In contrast, meta-analysis tools typically lose statistical power in such imbalanced or heterogeneous scenarios (details in the “Results” section).

## Results

We first verify *sPLINK* by comparing its results with those from aggregated analysis conducted with *PLINK* for all three association tests on a real GWAS dataset from the SHIP study [33]. We refer to this dataset as the *SHIP* dataset, which comprises the records of 3699 individuals with *serum lipase activity* as phenotype. The quantitative version represents the square root transformed serum lipase activity, while the dichotomous (binary) version indicates if the serum lipase activity of an individual is above or below the 75th percentile. The *SHIP* dataset contains around 5 million SNPs as well as sex, age, smoking status (current-, ex-, or non-smoker), and daily alcohol consumption (in g/day) as confounding factors (Table 1).

**Table 1:** Description of datasets

Dataset	*#* Samples	*#* SNPs	Adjustments	Phenotype
SHIP^a^	3699	∼5M	Sex, age, smoking status, daily alcohol consumption	SLA^b^, dichotomous (75th percentile, 934 cases, 2765 controls)
				SLA, quantitative, Mean ±SD^c^ 1.23 ±0.3
COPDGene^d^	5343	∼600K	Sex, age, smoking status, pack years of smoking	COPD^e^, dichotomous, (2811 cases, 2532 controls)
				FEV1^f^, quantitative, Mean ±SD 2.993 ±0.635
FinnGen	135,615	∼1M	Sex and age	Hypertension, dichotomous, (34,257 cases, 101,358 controls)

^a^Study of Health in Pomerania

^b^Serum lipase activity

^c^Standard deviation

^d^Genetic Epidemiology of chronic obstructive pulmonary disease

^e^Chronic obstructive pulmonary disease

^f^Forced expiratory volume in one second

We employ the binary phenotype for logistic regression and the chi-square test, and the quantitative phenotype for linear regression. We incorporate all four confounding factors in the regression models and no confounding factor in the chi-square test. We horizontally (sample-wise) split the dataset into four parts, simulating four different cohorts (Additional file 1: Table S1). *PLINK* computes the statistics for each association test using the whole dataset while *sPLINK* does it in a federated manner using the splits of the individual cohorts. To be consistent with *PLINK*, *sPLINK* calculates the same statistics as *PLINK* for the association tests.

**Fig. 3:**
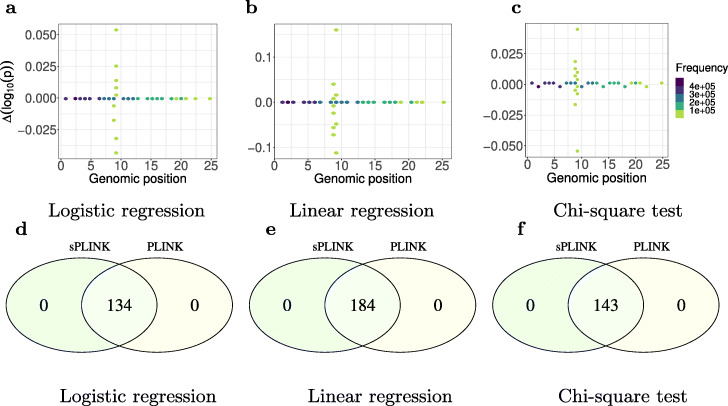
*Δlog*_10_(*p*-value) between *sPLINK* and *PLINK* as well as the set of SNPs identified by *sPLINK* and *PLINK* as significant for logistic regression (**a**, **d**), linear regression (**b**, **e**), and chi-square test (**c**, **f**), respectively. For most of the SNPs, the difference is zero, indicating that *sPLINK* gives the same *p*-values as *PLINK*. The negligible difference between *p*-values for the other SNPs can be attributed to differences in floating point precision. The spikes in some genomic positions are due to the strong association of the corresponding SNPs, which result in higher absolute error. *sPLINK* and *PLINK* also recognize the same set of SNPs as significant. Genomic positions (ticks in **a**–**c**) indicate chromosome numbers. The details of the experiments are available in Additional file 1: Table S1

We compute the difference between the *p*-values as well as the Pearson correlation coefficient (*ρ*) of *p*-values from *sPLINK* and *PLINK*. We use - *log*_10_(*p*-value) because the *p*-values are typically small and - *log*_10_(*p*-value) can be a better indicator of small *p*-value differences. According to Fig. 3a–c, the *p*-value difference is zero for most of the SNPs. We also observe that the maximum difference is 0.162 for a SNP in the linear regression. *sPLINK* and *PLINK* report 4.441×10^−16^ and 3.058×10^−16^ as *p*-values for the SNP, respectively. This negligible difference can be attributed to inconsistencies in floating point precision.

The correlation coefficient of *p*-values from *sPLINK* and *PLINK* for all three tests is $0.\overline {99}$, which is consistent with the results of *p*-value difference from Fig. 3a–c. We investigate the overlap of significantly associated SNPs between *sPLINK* and *PLINK*. We consider a SNP as significant if its *p*-value is less than 5×10^−8^ (genome-wide significance). *PLINK* and *sPLINK* recognize the same set of SNPs as significant (Fig. 3d–f). Notably, the identified SNPs, e.g. rs8176693 and rs632111, lying in genes ABO (intronic) and FUT2 (3-UTR), respectively, have also been implicated in a previous analysis of this dataset [34]. We also leverage the Bonferroni significance threshold (which is ≈1×10^−8^ for our tests) to compare the overlapping significant SNPs from *sPLINK* and *PLINK*. The results remain similar and the associated plot is available at Additional file 1: Fig. S1. These results indicate that *p*-values computed by *sPLINK* in a federated manner are the same as those calculated by *PLINK* on the aggregated data (ignoring negligible floating point precision error). In other words, the federated computation in *sPLINK* preserves the accuracy of the results of the association tests.

Next, we compare *sPLINK* with some existing meta-analysis tools, namely *PLINK*, *METAL*, and *GWAMA*. We leverage the *COPDGene* (non-hispanic white ethnic group) [35] and *FinnGen* (data release 3) [36] datasets. The *COPDGene* dataset has an equal distribution of case and control samples unlike the *SHIP* dataset. It contains 5343 samples (ignoring 1327 samples with missing phenotype value) and around 600K SNPs. We utilize chronic obstructive pulmonary disease (COPD) as the binary phenotype and include sex, age, smoking status, and pack years of smoking as confounding factors [37]. *FinnGen* is much larger dataset (in terms of sample size) compared to the *SHIP* and *COPDGene* datasets. It consists of 135,615 samples (ignoring 23 samples with missing phenotype value) and about 1 million SNPs. We use *Hypertension* as the (binary) phenotype and adjust for sex and age as confounding factors (Table 1).

**Fig. 4:**
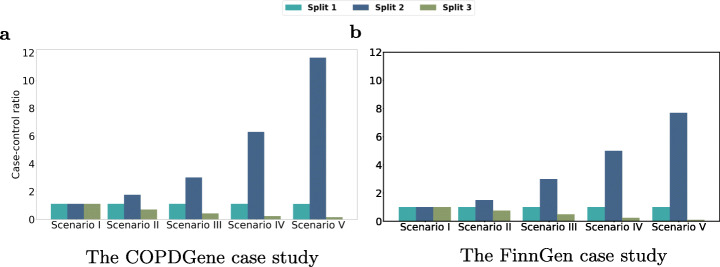
*Scenario I-V*: The case-control ratio is the same for all splits in the balanced scenario (I) while the splits have different case-control ratios in the imbalanced scenarios (II–V). All three splits have the same sample size in the *COPDGene* dataset as well as the balanced scenario in the *FinnGen* dataset. For the imbalanced scenarios in the *FinnGen* dataset, the splits have different sample sizes

**Fig. 5:**
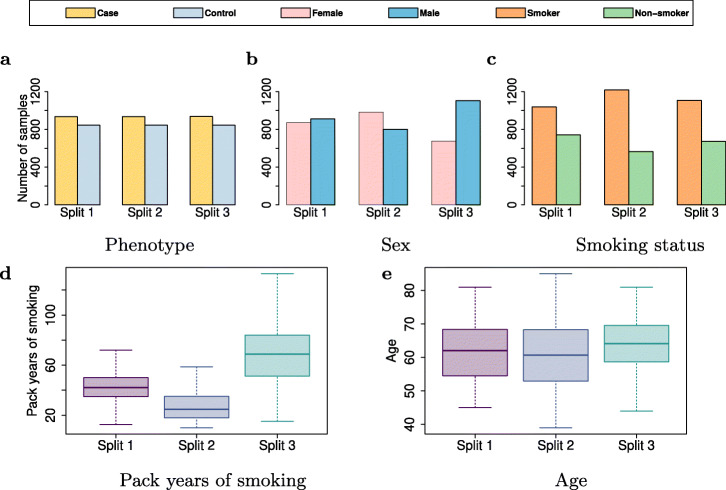
*Scenario VI (Heterogeneous Confounding Factor)* for the *COPDGene* case study: The phenotype distribution is the same and balanced; the values of smoking status and age are homogeneously distributed; the distribution of sex and pack years of smoking are slightly and highly heterogeneous across the splits, respectively

**Fig. 6:**
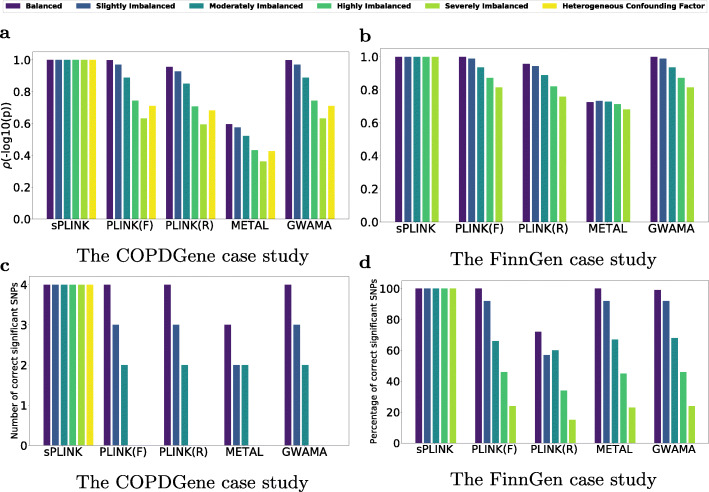
The Pearson correlation coefficient (*ρ*) of - *log*_10_(*p*-value) between each tool and aggregated analysis (**a**, **b**) and the number (**c**) and the percentage (**d**) of SNPs correctly identified as significant (true positives) by each tool. *F* and *R* stand for fixed-effect and random-effect, respectively. The details of the experiments are available in Additional file 1: Table S2, and Table S3

To simulate cross-study heterogeneity [38] on the *COPDGene* dataset, we consider six different scenarios: *Scenario I* (*Balanced*), *Scenario II* (*Slightly Imbalanced*), *Scenario III* (*Moderately Imbalanced*), *Scenario IV* (*Highly Imbalanced*), *Scenario V* (*Severely Imbalanced*), and *Scenario VI* (*Heterogeneous Confounding Factor*) (Figs. 4a and 5). In each scenario, we partition the dataset into three splits with the same sample size (more details in Additional file 1: Table S2). The distribution of all four confounding factors is homogeneous (similar) across the splits for the first five scenarios. The splits have the same (and balanced) case-control ratio in *Scenario I* and *Scenario VI* but their case-control ratio is different for the imbalanced scenarios (Fig. 4a). In *Scenario VI*, the values of two confounding factors (i.e. smoking status and age) are homogeneously distributed among the splits; however, the distribution of sex and pack years of smoking is slightly and highly heterogeneous across the splits, respectively (Fig. 5). We obtain the summary statistics (e.g. minor allele, odds ratio, and standard error) for each split to conduct meta-analyses. The results are then compared to the federated analysis employing *sPLINK*. Figure 6a shows the Pearson correlation coefficient of - *log*_10_(*p*-value) between each tool and the aggregated analysis for all six scenarios. Figure 6c depicts the number of SNPs correctly identified as significant by the tools (true positives).

According to Fig. 6a, the correlation of *p*-values between *sPLINK* and the aggregated analysis is ∼1.0 for all six scenarios, implying that *sPLINK* gives the same *p*-values as the aggregated analysis regardless of how phenotypes or confounding factors have been distributed across the cohorts. In contrast, the correlation coefficient for the meta-analysis tools shrinks with increasing imbalance/heterogeneity, indicating loss of accuracy. Figure 6c illustrates that *sPLINK* correctly identifies all four significant SNPs in all scenarios. In the balanced scenario, almost all meta-analysis tools perform well and recognize all significant SNPs. An exception is *METAL*, which misses one of them. However, they miss more and more significant SNPs as the phenotype imbalance across the splits increases. In the *Highly Imbalanced* and *Severely Imbalanced* scenarios, the meta-analysis tools cannot recognize any significant SNP. This is also the case if the distribution of some confounding factors becomes heterogeneous across the cohorts (*Scenario VI*). We checked the number of SNPs wrongly identified as significant by the tools (false positives) too. *sPLINK* has no false positive in any of the scenarios and the meta-analysis tools introduce zero or one false positive depending on the scenario.

To show that our findings on the *COPDGene* dataset also hold true for a much larger dataset, we repeat the simulations on the *FinnGen* dataset (more details in Additional file 1: Table S3). Similar to the *COPDGene* case study, we divide the dataset into three splits and define *Scenario I* to *Scenario V*, where the splits have the same case-control ratio (1.0) and sample size (22,838) as in *Scenario I* but different case-control ratios in the remaining scenarios (Fig. 4b); Unlike the *COPDGene* case study in which the sample size of the splits are equal for all scenarios including the imbalanced ones, the splits have different number of samples in the imbalanced scenarios of the *FinnGen* case study. For instance, split1, split2 and split3 have 22,838, 12,561, and 99,345 samples in *Scenario V*, respectively (a split with lower case-control ratio has larger sample size). It implies that the aggregated datasets have different number of samples in the scenarios, and as a result, there are different set of significant SNPs in each scenario of the *FinnGen* case study (total of 110, 116, 199, 304, and 446 significant SNPs in *Scenario I* to *Scenario V*, respectively).

Figures 6b and 6d illustrate the Pearson correlation coefficient and percentage of correctly identified significant SNPs for each scenario on the *FinnGen* case study, respectively. According to Fig. 6b, the correlation coefficient diminishes for the meta-analysis tools as the scenario becomes more and more imbalanced. This is also the case for the percentage of the SNPs correctly identified as significant by each meta-analysis tool (Fig. 6d). These results are consistent with those from the *COPDGene* case study. Moreover, we observed that the meta-analysis tools report high number of false positives (14–88) in *Scenario IV*. Thus, the limitations of meta-analysis tools towards class imbalance observed in the *COPDGene* dataset can be reproduced on a large dataset. However, sPLINK always provides the same results as PLINK with the aggregated analysis (the “Methods” section, Figs. 3 and 6a, c).

We also leverage the Spearman correlation to check whether or not the meta-analysis tools maintain the ordering of significance compared to the aggregated analysis. Our results show that this is not the case, and the Spearman correlation values for the meta-analysis tools reduce as the phenotype imbalance across the splits increases, similar to the results from Fig. 6, where the Pearson correlation is used. The corresponding plot can be found in Additional file 1: Figure S2.

Table 2 shows a concise comparison between *sPLINK* and the state-of-the-art approaches. Unlike *PLINK*, *sPLINK* is privacy-aware, where the private data never leaves the cohorts. *sPLINK* is also robust against the imbalance/heterogeneity of phenotype/confounding factor distributions across the cohorts. *sPLINK* always delivers the same *p*-values as aggregated analysis and correctly identifies all significant SNPs independent of the phenotype or confounding factor distribution in the cohorts. In contrast, meta-analysis tools lose their statistical power in imbalanced phenotype scenarios, missing some or all significant SNPs. This is also the case if the phenotype distribution is balanced but the values of confounding factor(s) have heterogeneously been distributed across the datasets. Compared to the existing SMPC/HE-based approaches, *sPLINK* is computationally efficient and supports multiple association tests including chi-square and linear/logistic regression. *sPLINK* provides enhanced privacy by hiding the model parameters of each cohort from the third parties while federated learning-based frameworks such as GLORE reveal them to the server.

**Table 2:** Comparison between *sPLINK* and the state-of-the-art approaches

Tool/Study	Privacy-aware	Robust to heterogeneity	Computationally efficient	Linear regression	Logistic regression
PLINK	✗	✓	✓	✓	✓
Meta-analysis	✓	✗	✓	✓	✓
Kamm et al. [17]	✓	✓	✗	*	✗
Cho et al. [18]	✓	✓	✗	*	✗
Morshed et al. [23]	✓	✗	✗	✓	✗
Kim et al. [24]	✓	✗	✗	✗	✓
GLORE [28]	✓	✓	✓	✗	✓
sPLINK	✓	✓	✓	✓	✓

*The study supports the Cochran–Armitage test, which is computationally comparable to linear regression

Finally, we measure the runtime and network bandwidth usage of *sPLINK* for each association test using the COPDGene dataset partitioned into three splits of the same sample size. We use *COPD* in chi-square as well as logistic regression and *FEV1* in linear regression as phenotype. We include age, sex, smoking status, and pack years of smoking as confounding factors only for the regression tests. The server and WebApp packages are installed on a physical machine located at *Freising* (*Germany*) while the compensator is running on a machine at *Odense* (*Denmark*). Three commodity laptops located at *Munich* or *Freising* are running the client package and host the splits. They communicate with the server and compensator through the Internet. The system specification of the machines and laptops as well as the details of the experiments can be found in Additional file 1: Table S4 and S5.

**Fig. 7:**
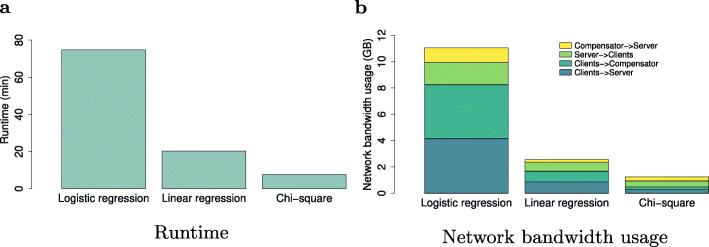
Runtime and network bandwidth consumption of *sPLINK*. Logistic regression is the most time-consuming association test and exchanges the highest traffic over the network due to the iterative nature of the algorithm. The experimental setup can be found in Additional file 1: Table S5

Figure 7a plots the *sPLINK’s* runtime for each association test. *sPLINK* computes the results for chi-square, linear regression, and logistic regression in 8 min, 20 min, and 75 min, respectively. Sending parameters from the clients to the server and compensator contributes the most in sPLINK’s runtime. Compared to Kamm et al. [17], *sPLINK* is almost 13 times faster for chi-square test (8 min vs. 110 min^1^) with less powerful hardware, larger sample size (5343 vs. 1080), and more number of SNPs (∼580K vs. ∼263K).

Figure 7b depicts the network usage of *sPLINK*. The clients, server, and compensator exchange total of 0.967 GB, 2.49 GB, and 11.06 GB traffic in chi-square, linear regression, and logistic regression, respectively. Logistic regression has higher volume of traffic exchange because the computation of beta coefficients are performed in an iterative fashion. A fair comparison between *sPLINK* and SMPC-based frameworks from the network communication aspect is tricky. However, in general, (hybrid) federated learning-based approaches consume more network bandwidth than SMPC-based ones.

We also conduct a set of experiments to investigate how the runtime and network bandwidth consumption of *sPLINK* change with varying number of samples, SNPs, and clients. The results demonstrate that the traffic exchanged over the network is independent of the sample size and linearly increases with the number of SNPs and clients (as expected). Moreover, runtime is not affected much by the sample size thanks to the multi-threading capability of *sPLINK*’s client package, and linearly/non-linearly increases with the number of SNPs/clients. The corresponding plots are available in Additional file 1: Fig. S3, S4, and S5.

## Discussion

We first provide a general discussion on the privacy of the existing tools for collaborative GWAS including *sPLINK*. To be more accurate, we draw a distinction between the privacy-aware and privacy-preserving definitions [39]. In a privacy-aware approach, it is not required to share the private data with a third party. A privacy-aware approach is privacy-preserving if the approach offers a privacy guarantee that captures the privacy risk associated with individual samples in the dataset. Given that, meta-analysis, SMPC, HE, federated learning, and hybrid federated learning based on SMPC are privacy-aware because they do not share the raw data with a third party. In meta-analysis/federated learning, the summary statistics/model parameters of each cohort are shared with a third party. In SMPC-based hybrid federated learning, the aggregated (global) parameters are revealed to the server and cohorts. These approaches, including HE and SMPC, reveal the final model too. However, these methods are not privacy-preserving because none of them provides a privacy guarantee indicating to what extent the revealed information leaks the private data of a particular sample in the dataset. To our knowledge, differential privacy (DP) [40] and DP-based hybrid federated learning can offer such a guarantee at the cost of the utility of the model and are considered as privacy-preserving approaches.

While privacy-aware approaches do not offer a privacy guarantee, they might provide stronger/weaker privacy compared to each other based on the amount and nature of the information they share with third parties. For instance, HE-based methods provide stronger privacy because they only reveal the final model (results) while other privacy-aware approaches disclose not only the final results but also other information such as summary statistics or local parameters. Similarly, *sPLINK* provides enhanced privacy in comparison with existing federated learning based tools such as GLORE. This is because GLORE discloses the local parameters of each cohort to the server, which is not revealed in *sPLINK*.

*sPLINK* is a privacy-aware tool, assuming honest-but-curious server, compensator, and clients, which (I) follow the protocol as it is; for instance, the server always sends the global beta values resulted from the aggregation but not the beta values tampered with such as all zeros to the clients, and (II) do not collude with each other, e.g. the compensator never shares the individual noise values of the clients with the server and similarly, the server does not send the noisy local parameters to the compensator, but (III) they try to reconstruct the raw data using the model parameters. Additionally, (IV) there are at least three different cohorts participating in the study, and their client components as well as the server and compensator components are running in separate physical machines.

Given these assumptions, we discuss the privacy of the masking mechanism of *sPLINK* (inherited from *HyFed*) for the supported association tests. To this end, we use the information theoretic criterion called *mutual information* between two random variables *X* and *Y* [30, 41]:

$$I(X, Y) = H(X) - H(X|Y) $$

where *H*(*X*) and *H*(*X*|*Y*) indicate the entropy of *X* and the conditional entropy of *X* given *Y*, respectively. The mutual information measures (in bits) the decrease in uncertainty about *X* having the knowledge of *Y*. In *sPLINK*, the noisy local parameter $M^{\prime }_{L}$ is a secret share from the local parameter *M*_*L*_ (the secret), and random variables *X* and *Y* indicate the distributions of *M*_*L*_ and $M^{\prime }_{L}$, respectively.

The local parameter *M*_*L*_ of a client is either a non-negative integer (e.g. sample count, allele count, or contingency table) or floating-point number (e.g. Hessian or covariance matrix) in the association tests. For non-negative integers, *sPLINK* capitalizes on *additive secret sharing* based on *modular arithmetic* over the finite field $\mathbb {Z}_{p}$= {0, 1, *p*−1}, in which *p* is a *prime* number [13]. For floating-point numbers, *sPLINK* employs *real value secret sharing* based on Gaussian (Normal) distribution [42, 43] (more details in “Methods” section).

For non-negative integers, noise *N*_*L*_ is generated from a uniform distribution over $\mathbb {Z}_{p}$, and $M^{\prime }_{L}$ is the modular addition of *M*_*L*_ and *N*_*L*_: $M^{\prime }_{L}$ = (*M*_*L*_ + *N*_*L*_) mod *p*. For this scheme, it has been shown that the knowledge of *Y* (noisy local parameter) provides no information about *X* (local parameter), which means the mutual information between them is zero: *I*(*X*,*Y*)=0 [13, 16]. Notice that this is the case for any value of prime number *p*.

For floating-point numbers, noise *N*_*L*_ is generated using Gaussian distribution with variance of $\sigma ^{2}_{N}$. Assuming that the variance of *X* is $\sigma ^{2}_{M_{L}}$, the mutual information between *X* and *Y* is maximum if *Y* follows the Gaussian distribution (variance $\sigma ^{2}_{M_{L}} + \sigma ^{2}_{N}$) [43]. Thus, the upper bound on the mutual information between *X* and *Y* is:

$$ I(X, Y) = \frac{1}{2}\log_{2}(1 + \frac{\sigma^{2}_{M_{L}}}{\sigma^{2}_{N}}) $$

That is, the amount of reduction in uncertainty about the local parameters having the knowledge of the noisy local parameters depends on the relative variance of the corresponding distributions. Therefore, using larger values for variance in the Gaussian random generator will provide lower information leakage. The value of mean for the Gaussian random generator does not remarkably impact the privacy and can be set to zero [43], which is the case for *sPLINK*. The default value of $\sigma ^{2}_{N}$ is 10^12^ for *sPLINK*, which is large enough for typical GWAS, but it can be set to higher values if needed to ensure that $\frac {\sigma ^{2}_{M_{L}}}{\sigma ^{2}_{N}}$ remains small.

Notice that although *sPLINK* significantly enhances the privacy of data compared to existing federated learning tools by hiding the local parameters of clients from a third party, it does not eliminate the possibility of data reconstruction using the aggregated parameters or final results. For example, the *X*^*T*^*X* parameter (covariance matrix) in the linear regression algorithm can be exploited to determine the sex of the patients if the total number of samples across all cohorts is comparable to the number of the confounding factors. However, for a reliable GWAS study, the total sample size is considerably larger than the number of confounding factors, and therefore, the reconstruction of the cohorts’ private data from the aggregated parameters can be difficult (but still possible) in practice. A similar argument is also applicable to meta-analysis approaches, which reveal the summary statistics of each cohort to a third party.

The value of prime number *p* impacts the correctness of the masking mechanism. To ensure the correctness, overflow must not occur in $\sum _{i=1}^{i=K} N_{L_{i}}$ and $\sum _{i=1}^{i=K} M^{\prime }_{L_{i}}$ calculations, and $\sum _{i=1}^{i=K} M_{L_{i}} < p$. *sPLINK* uses the default value of *p* = 2^54^−33, which is the largest prime number than can fit in 54-bit integer. A higher value of *p* can be employed to handle larger integer values but at the expense of a lower number of clients [30]. Likewise, too large values of variance $\sigma ^{2}_{N}$ (e.g. 10^30^) can impact the precision of the results. With default values of *p* and $\sigma ^{2}_{N}$, however, our experiments indicate that there are no statistically significant differences between the results from *sPLINK* with and without the masking mechanism for all three association tests (the experimental setup of Fig. 7 is used in the experiments).

*sPLINK* currently supports chi-square and linear/logistic regression tests, but it can be extended to compute other useful statistics in GWAS such as minor allele frequency (MAF), Hardy-Weinberg equilibrium (HWE), and linkage disequilibrium (LD) between SNPs in a privacy-aware manner. The federated computation of the aforementioned statistics in *sPLINK* is expected to be straightforward because they are based on the allele frequencies, and *sPLINK* already calculates the minor and major allele counts in the *Non-missing count* step of its computational workflow (the “Methods” section). Moreover, population stratification using the principal component analysis (PCA) will be addressed in the future version of *sPLINK* due to the complexity of the problem. *sPLINK*’s implementation of the association tests is horizontally-federated, where the datasets have different samples but the same features (i.e. SNP and confounding factors). However, correcting for population structure using *sPLINK* requires a vertically-federated [44] PCA algorithm because the eigenvectors should be computed from the sample by sample covariance matrix, and therefore, the samples and features swap roles in the federated PCA (SNPs are considered as samples and patients as features) [45]. Vertical federated learning algorithms are still understudied, and they are considered more complicated than the horizontal algorithms.

Additionally, the federated PCA algorithm should be an iterative, randomized algorithm [46] so that it can handle large GWAS datasets with a practical amount of main memory. The iterative nature of the algorithm will present network and runtime challenges because it might need dozens or hundreds of iterations and exchange huge traffic over the network to converge to the final eigenvectors. From the privacy perspective, a recent study [45] demonstrates that even if we assume the federated PCA and linear regression algorithms individually provide perfect privacy, federated population stratification in GWAS, where the eigenvectors are used as the confounding factors in the association test, does not necessarily offer perfect privacy. Consequently, the server can reconstruct the SNP or binary confounding factor values in polynomial time. To tackle this issue, they suggested that the final eigenvectors should be computed at the clients and the model parameter values should be hidden from the server. The federated population stratification in *sPLINK* should be implemented taking into account those suggestions.

We showed that *sPLINK* is robust against an important source of data heterogeneity, namely the heterogeneous distribution of the phenotype or confounding factor values across the distributed datasets of the cohorts. Population heterogeneity across the cohorts is another source of data heterogeneity in GWAS, which is commonly tackled by population stratification using the PCA algorithm. *sPLINK* currently does not address this kind of data heterogeneity but the future versions of the tool will support population stratification to this end.

## Conclusions

We introduce *sPLINK*, a user-friendly, hybrid federated tool for GWAS. *sPLINK* enhances the privacy of the cohorts’ data without sacrificing the accuracy of the test results. It supports multiple association tests including chi-square, linear regression, and logistic regression. *sPLINK* is consistent with *PLINK* in terms of the input data formats and results. We compare *sPLINK* to aggregated analysis with *PLINK* as well as meta-analysis with *METAL*, *GWAMA*, and *PLINK*. While *sPLINK* is robust against the heterogeneity of phenotype or confounding factor distributions across separate datasets, the statistical power of the meta-analysis tools is declined in imbalanced/heterogeneous scenarios. We argue that *sPLINK* is easier to use for collaborative GWAS compared to meta-analysis approaches thanks to its straightforward functional workflow. We also show that *sPLINK* achieves practical runtime, in order of minutes or hours, and acceptable network usage. *sPLINK* is an open-source tool and its source code is publicly available under the Apache License Version 2.0. *sPLINK* is a novel and robust alternative to meta-analysis, which performs collaborative GWAS in a privacy-aware manner. It has the potential to immensely impact the statistical genetics community by addressing current challenges in GWAS including cross-study heterogeneity and, thus, to replace meta-analysis as the gold standard for collaborative GWAS.

## Methods

*Federated learning* [14, 15] is a type of distributed learning, where multiple cohorts collaboratively learn a joint (global) model under the orchestration of a central server [47]. The cohorts never share their private data with the server or the other cohorts. Instead, they extract local parameters from their data and send them to the server. The server aggregates the local parameters from all cohorts to compute the global model parameters (or global results), which in turn, are shared with all cohorts. While federated learning is privacy-aware, where the private data of the cohorts is not shared with the server, studies [48, 49] have shown that for some models such as deep neural networks, the raw data can be reconstructed from the parameters shared by the cohorts.

To improve the privacy of federated learning, privacy-enhancing technologies (PETs) such as DP, HE, or SMPC can be combined with federated learning to avoid revealing the original values of the local parameters to third parties including the server [50]. DP-based hybrid federated learning approaches can provide a privacy guarantee but their final results might be considerably impacted by the random noise employed for the perturbation of the model. HE-based aggregation methods can incur remarkable computational overhead because they require the cohorts to encrypt/decrypt the local/global model parameters and the server to perform the aggregation over the encrypted parameters. SMPC-based hybrid federated learning methods [30, 51] increase the network bandwidth usage but does not adversely affect the final results. *HyFed* is an open-source hybrid federated framework, which combines federated learning with additive secret sharing-based SMPC to enhance the privacy of the federated algorithms while preserving the utility (performance) of the global model. HyFed provides a generic API (application programming interface) to develop federated machine learning algorithms. It supports the federated mode of operation, where different components of the framework can be installed in separate physical machines and securely communicate with each other through the Internet.

*sPLINK* implements a hybrid federated approach using the *HyFed* API to enhance the privacy of data. *sPLINK* works with distributed GWAS data, where samples are individuals and features are SNPs and categorical or quantitative phenotypic variables. While the samples are different across the cohorts, the feature space is the same because *sPLINK* only considers SNPs and phenotypic variables that are common among all datasets (horizontal or sample-based federated learning)[44]. The client package of *sPLINK* is installed on the local machine of each cohort with access to the private data. The compensator is running in a separate machine. sPLINK’s server and WebApp packages are installed on a central server.

In *sPLINK*, the original values of the parameters computed from the private data in one cohort is not revealed to the server, compensator, or other cohorts, improving the privacy of the cohorts’ data. *sPLINK* provides the chunking capability to handle large datasets containing millions of SNPs. The chunk size (configured by the coordinator) specifies how many SNPs should be processed in parallel. Larger chunk sizes allow for more parallelism, and therefore less running time in general but require more computational resources (e.g. CPU and main memory) from the local machines of the cohorts, the server, and compensator. *sPLINK*’s client package is multi-threaded, where the number of cores is configurable by the participants. This makes the computation of the model parameters in the cohorts very fast, especially for large datasets. While we provide a readily usable web service running at *exbio server* (https://exbio.wzw.tum.de/splink) and online compensator at *compbio server* (https://compensator.compbio.sdu.dk), the server, WebApp, and compensator packages can, of course, be deployed on customized physical machines.

The *functional workflow* of *sPLINK* is comprised of the following steps:

1. **Project creation**: The coordinator creates the project (new study) through the Web interface. To this end, she/he first specifies the project name, association test name, chunk size, and the list of confounding features (only for regression tests), and then, generates a unique project token for each cohort.
2. **Cohort invitation**: The coordinator sends the project ID (automatically generated) and token to each participant (a human entity interacting with the client package in a cohort) through a secure channel such as email for inviting the cohorts to the project.
3. **Cohort joining**: The participants use their corresponding username, password, project ID, and token to join the project. After joining, they can view the general information of the project such as the coordinator, server/compensator name/URL, and etc. If they agree to proceed, they choose the dataset they want to employ in the study. To be consistent with *PLINK*, *sPLINK* supports *.bed* (value of SNPs), *.fam* (sample IDs as well as sex and phenotype values), *.bim* (chromosome number, name, and base-pair distance of each SNP), *.cov* (value of confounding factors), and *.pheno* (phenotype values that should be used instead of those in *.fam* file) file formats as specified in the *PLINK* manual [52]. For linear regression, phenotype values must be quantitative while for logistic regression and chi-square, phenotype values have to be binary (control/case are encoded as 1/2).
4. **Federated computation**: In *sPLINK*, the association test results are computed by the client package (running on the local machines of cohorts), server package (running in the central server), and compensator (running in its own machine) in a federated manner. The computation is iterative and consists of six general steps:
  - **Get global parameters**: All clients obtain the required global parameters *M*_*G*_ from the server.
  - **Compute local parameters**: Each client *i* computes the local parameters $M_{L_{i}}$ using the local data and global parameters.
  - **Mask local parameters**: Each client *i* generates random noise $N_{L_{i}}$ with the same shape as $M_{L_{i}}$, and masks $M_{L_{i}}$ with $N_{L_{i}}$ to obtain the noisy local parameters $M^{\prime }_{L_{i}}$.
  - **Share noisy local parameters and noise**: Each client *i* shares $M^{\prime }_{L_{i}}$ and $N_{L_{i}}$ with the server and compensator, respectively.
  - **Aggregate noise**: The compensator computes the aggregated noise *N* given the noise values from the clients and sends the aggregated noise *N* to the server.
  - **Compute global parameters**: The server calculates (unmasks) the global parameters given the noisy local parameters and the negative of the aggregated noise.
5. **Result download**: The final results are automatically downloaded for the cohorts but the coordinator needs to download them manually through the web interface. Similar to *PLINK*, *sPLINK* reports minor allele name (*A1*) and *p*-value (*P*) for all three association tests, chi-square (*CHISQ*), odds ratio (*OR*), minor allele frequency in cases (*F*_*A*), and minor allele frequency in controls (*F*_*U*) for chi-square test, and the number of non-missing samples (*NMISS*), beta (*BETA*), and t-statistic (*STAT*) for linear and logistic regression tests.

*sPLINK* inherits its masking mechanism from *HyFed*, which masks the local parameters with non-negative integer and floating-point values in different ways. For a local parameter with a non-negative integer value, *sPLINK* considers a finite field $\mathbb {Z}_{p}$= {0, 1, *p*−1} (*p* is a *prime* number) [13], where each client *i* generates a uniform random integer from $\mathbb {Z}_{p}$ as noise $N_{L_{i}}$ and masks its local parameter $M_{L_{i}}$ with $N_{L_{i}}$ by performing the *modular addition* over $\mathbb {Z}_{p}$: $M^{\prime }_{L_{i}}$ = ($M_{L_{i}}$ + $N_{L_{i}}$) mod *p*. Notice that $M_{L_{i}},N_{L_{i}},M^{\prime }_{L_{i}} \in \mathbb {Z}_{p}$. For $M_{L_{i}}$ with a floating-point value, each client *i* generates noise $N_{L_{i}}$ using Gaussian random generator with zero-mean and variance $\sigma ^{2}_{N}$, and masks $M_{L_{i}}$ with $N_{L_{i}}$ using the ordinary addition: $M^{\prime }_{L_{i}}$ = $M_{L_{i}}$ + $N_{L_{i}}$.

The compensator computes the aggregated noise *N* by taking sum over the noise values of the clients using the modular or ordinary addition depending on the data type of the noise: if $N_{L_{i}}$ is non-negative integer, then *N* = ($\sum _{i=1}^{i=K} N_{L_{i}}$) mod *p*; if $N_{L_{i}}$ is floating-point type, then *N* = $\sum _{i=1}^{i=K} N_{L_{i}}$. To calculate the global parameters with non-negative integer values, the server first computes the aggregated noisy parameter by taking sum over the noisy local parameters using the modular addition, and then subtracts the aggregated noise from the aggregated noisy parameter using the modular subtraction: *M*_*G*_ = ((($\sum _{i=1}^{i=K} M^{\prime }_{L_{i}}$) mod *p*) - *N*) mod *p*. For model parameters with floating-point values, the server adds up the noisy local parameters and the negative of the aggregated noise using the ordinary addition: *M*_*G*_ = $\sum _{i=1}^{i=K} M^{\prime }_{L_{i}} - N$.

The *computational workflow* of *sPLINK* involves seven steps common among all association tests as well as a couple of steps specific to each association test (Fig. 8). In the first three steps (i.e. *Init*, *SNP name*, and *Allele name*) as well as the sixth step (*Minor allele*), the clients only communicate with the server, where the name of the SNPs and alleles (which are not considered private) are directly shared with the server. In the remaining steps, the compensator is involved and clients mask the local parameters with noise to hide their original values from the server. The formulas associated with the steps indicate how the clients compute local parameters and how the server calculates the global parameters using the noisy local parameters of the clients and the aggregated noise from the compensator. In the following, we provide an overview of each step:

1. **Init**: Each client *i* opens the files of the dataset selected by the participant to be employed in the study and creates its phenotype vector (*Y*_*i*_) and feature matrix (*X*_*i*_), which includes the value of SNPs and confounding factors. It is worth noting that there is a separate feature matrix for each SNP but the phenotype vector is the same for all SNPs. Assume a dataset containing three SNPs named *SNP1*, *SNP2*, and *SNP3* and *age* and *sex* as confounding features. There will be three different feature matrices, one feature matrix per SNP. For instance, the feature matrix of *SNP1* has three columns including *SNP1*, *age*, and *sex* values. Phenotype vector and feature matrix are the private data of the cohorts. They cannot be shared with the server, compensator, or the other cohorts. The aggregation process in the server just makes sure that all clients successfully initialized their data.

**Fig. 8:**
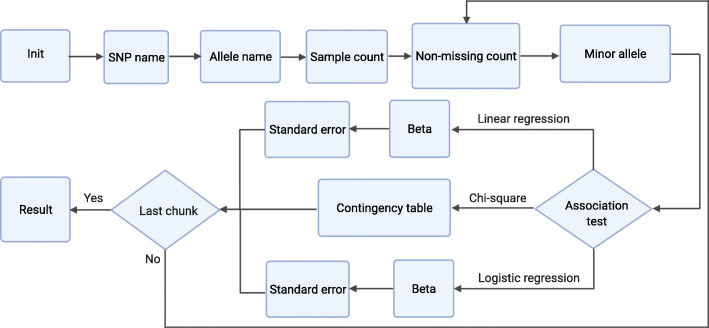
Computational workflow of *sPLINK*: The first six steps and the last step are common among all association tests. Contingency table is specific to the chi-square test while Beta and Standard error are regression test related steps
2. **SNP name**: Each client shares the SNP names with the server. In the aggregation process, the server computes the intersection of all SNP names. Only common SNPs are considered in the computation of the association test results.
3. **Allele name**: Each client sends the allele names (e.g. G,A) of each SNP to the server. In the aggregation process, the server ensures that all cohorts employ the same allele names for the SNPs. Notice that the clients sort the allele names to avoid revealing which one is minor or major allele.
4. **Sample count**: Each client *i* calculates its local sample count *T*_*i*_ (number of samples in its dataset including missing samples, which is the size of vector *Y*_*i*_). The server computes the corresponding global sample count: *T* = $(((\sum _{i=1}^{i=K} T^{\prime }_{i}) \mod $*p*) - *N*_*T*_) mod *p*, where $T^{\prime }_{i}$ is the noisy local sample count of client *i*: $T^{\prime }_{i}$ = (*T*_*i*_+*N*_*i*_) mod *p* and *N*_*T*_ is the aggregated noise from the compensator: *N*_*T*_ = ($\sum _{i=1}^{i=K} N_{i}) \mod $*p*.
5. **Non-missing count**: In this step, SNPs are split into chunks which can be processed in parallel. The chunking capability is provided to handle very large datasets containing millions of SNPs. The clients compute the non-missing sample count by filtering out the missing samples (value of -9 is considered as missing). Likewise, they calculate the local allele count by counting the number of alleles in each SNP. In the aggregation process, the server computes the global non-missing sample count (*n*) and allele count using the corresponding noisy parameters and the aggregated noise similar to the sample count step. Finally, the server determines the global minor allele based on the values of the global allele counts.
6. **Minor allele**: The clients compare their local minor allele with the global minor allele. If they are the same, they do nothing. Otherwise, they update the mapping of SNP values read from.bed file. Each SNP value can be 0, 1, 2, or 3 (missing value). These values are encoded based on the minor allele name. If the minor allele is changed, the value of the SNP needs to be swapped if it is 0 or 2. Thus, if a client’s minor allele is different from global minor allele, it inverses the mapping of SNP values (0→2 and 2→0). The aggregation in the server makes sure that all clients successfully completed this step.
7. **Association test specific steps**: In the following, we elaborate on the steps specific to each association test. Regarding regression tests, *sPLINK* implements the federated versions of ordinary least squares linear regression and Newton-Raphson method based logistic regression.
  **Chi-square**: The only test-specific step for the chi-square test is *Contingency table*, where each client *i* computes its local contingency table containing minor allele frequency for cases (*t*_*i*_), minor allele frequency for controls (*r*_*i*_), major allele frequency for cases (*q*_*i*_), and major allele frequency for controls (*s*_*i*_). The server aggregates the noisy contingency tables from the clients ($t^{\prime }_{i}, r^{\prime }_{i}, q^{\prime }_{i}$, and $s^{\prime }_{i}$ are the elements of the table) and the corresponding aggregated noise from the compensator (*N*_*t*_,*N*_*r*_,*N*_*q*_, and *N*_*s*_) to compute the global (observed) contingency table (Table 3). It also calculates the expected contingency table based on the observed contingency table (Table 4).

**Table 3:** Global (observed) contingency table

	Minor allele	Major allele	Total
**Case**	*t* = $(((\sum _{i=1}^{i=K} T^{\prime }_{i}) \mod $*p*) - *N*_*t*_) mod *p*	*q* = ((($\sum _{i=1}^{i=K} q^{\prime }_{i}) \mod $*p*) - *N*_*q*_) mod *p*	*t* + *q*
**Control**	*r* = $(((\sum _{i=1}^{i=K} r^{\prime }_{i}$) mod *p*) - *N*_*r*_) mod *p*	*s* = ((($\sum _{i=1}^{i=K} s^{\prime }_{i}) \mod $*p*) - *N*_*s*_) mod *p*	*r*+*s*
**Total**	*t* + *r*	*q* + *s*	2n

**Table 4:** Expected contingency table

	Minor allele	Major allele
**Case**	$\frac {(t+q) \times (t+r)}{2n}$	$\frac {(t+q) \times (q+s)}{2n}$
**Control**	$\frac {(r+s) \times (t+r)}{2n}$	$\frac {(r+s) \times (q+s)}{2n}$
  Given the observed contingency table (*O*) and the expected contingency table (*E*), the server computes odds ratio (OR), *χ*^2^, and *p*-value (*P*) as follows:
  1 $$ \text{OR} = \frac{t \times s}{q \times r}  $$
  2 $$ \chi^{2} = \sum \frac{(E - O)^{2}}{E}  $$
  3 $$ P = 1 - F_{t}(\chi^{2}, 1)  $$
  where *F*_*t*_ is the cumulative distribution function (CDF) of *χ*^2^ distribution (degree of freedom is 1).
  **Linear regression**: *Beta* and *Standard error* are two steps specific to linear regression test. In the *Beta* step, each client *i* computes $X_{i}^{T}X_{i}$ and $X_{i}^{T}Y_{i}$, where $X_{i}^{T}$ is the transpose of *X*_*i*_. In the aggregation process, the server performs the following calculations (*K* is the number of clients):
  4 $$ X^{T}X = \sum_{i=1}^{i=K} (X_{i}^{T}X_{i})^{\prime} - N_{X^{T}X}  $$
  5 $$ X^{T}Y = \sum_{i=1}^{i=K} (X_{i}^{T}Y_{i})^{\prime} - N_{X^{T}Y}  $$
  6 $$ \beta = (X^{T}X)^{-1}(X^{T}Y)  $$
  where $(X_{i}^{T}X_{i})^{\prime }$ and $(X_{i}^{T}Y_{i})^{\prime }$ are the noisy local parameters from the clients, $\phantom {\dot {i}\!}N_{X^{T}X}$ and $\phantom {\dot {i}\!}N_{X^{T}Y}$ are the corresponding aggregated noise from the compensator, and ()^−1^ indicates the inverse matrix.
  In the *Standard error* step, each client *i* calculates the local sum square error (SSE) *E*_*i*_ by having the global *β* vector.
  7 $$ \hat{Y_{i}} = X_{i} \beta  $$
  8 $$ E_{i} = \sum (Y_{i} - \hat{Y_{i}})^{2}  $$
  and then the server calculates the global standard error vector (SE) as follows:
  9 $$ E = \sum_{i=1}^{i=K}{{E^{\prime}_{i} - N_{E}}}  $$
  10 $$ \text{VAR} = (\frac{E}{n-m-1})(X^{T}X)^{-1}  $$
  11 $$ \text{SE} = \sqrt{\text{diag}(\text{VAR})}  $$
  where $E^{\prime }_{i}$ and *N*_*E*_ are the noisy SSE values and the corresponding aggregated noise, respectively; *n* is the global non-missing sample count, *m* is the number of features (1 + number of confounding factors), and *diag* is the main diagonal of the matrix.
  Given the standard error vector, the server computes the *T statistic* (*T*) and *p-value* (*P*) as follows:
  12 $$ T = \frac{\beta}{\text{SE}}  $$
  13 $$ \text{DF} = n - m - 1  $$
  14 $$ P = 2 \times (1 - F_{t}(|T|, \text{DF}))  $$
  in which *DF* is degree of freedom and *F*_*t*_ is the CDF of T distribution.
  **Logistic regression**: Similar to linear regression, logistic regression has two specific steps: *Beta* and *Standard error*. However, the *Beta* step is iterative in logistic regression (maximum number of iterations is specified by the coordinator and its default value is 20). In each iteration, each client *i* computes local gradient (∇_*i*_), Hessian matrix (*H*_*i*_) and log-likelihood (*L*_*i*_) as follows:
  15 $$ \hat{Y_{i}} = \frac{1}{1+e^{-X_{i}\beta}}  $$
  16 $$ \nabla_{i} = X_{i}^{T} (Y_{i}-\hat{Y_{i}})  $$
  17 $$ H_{i} = (X_{i}^{T} \circ (\hat{Y_{i}} \circ (1-\hat{Y_{i}}))^{T}) X_{i}  $$
  18 $$ L_{i} = \sum (Y_{i} \circ \log\hat{Y_{i}} + (1-Y_{i}) \circ \log(1-\hat{Y_{i}}))  $$
  where *β* is the global beta vector from the previous iteration and ∘ indicates element-wise multiplication.
  The server aggregates the noisy local gradients ($\nabla _{i}^{\prime }$), Hessian matrices ($H_{i}^{\prime }$) and log-likelihood values ($L_{i}^{\prime }$) from *K* clients and the associated aggregated noise values *N*_∇_,*N*_*H*_,*N*_*L*_ as follows:
  19 $$ \nabla = \sum_{i=1}^{i = K} \nabla_{i}^{\prime} - N_{\nabla}  $$
  20 $$ H = \sum_{i=1}^{i = K} H_{i}^{\prime} - N_{H}  $$
  21 $$ L = \sum_{i=1}^{i = K} L_{i}^{\prime} - N_{L}  $$
  Then, it updates the *β* values accordingly:
  22 $$ \beta_{\text{new}} = \beta_{\text{old}} + {H}^{-1} \nabla  $$
  where *β*_old_ is the *β* value from the previous iteration. The server also compares the newly computed log-likelihood value (L) with the one from previous iteration (*L*_old_). If their difference is less than a pre-specified threshold, *β* values converged, and therefore, it stops updating beta.
  In the *Standard error* step, the server shares the global *β* values with the clients. Each client *i* computes its local Hessian matrix (*H*_*i*_) using the global *β*. The server gets the noisy local Hessian matrices from *K* clients and the aggregated noise from the compensator and applies the following formula to obtain the global standard error vector (SE):
  23 $$ \text{SE} = \sqrt{\text{diag}\bigg(\big(\sum_{i=1}^{i=K}{H_{i}^{\prime} - N_{H}}\big)^{-1}\bigg)}  $$
  Having standard error values, the server calculates *T* statistics and *p*-value (*P*) as follows:
  24 $$ T = \frac{\beta}{\text{SE}}  $$
  25 $$ P = 1 - F_{t}(|T|^{2}, 1)  $$
  where *F*_*t*_ is CDF of *χ*^2^ distribution (degree of freedom is 1).
8. **Result**: The computation of association test results have been completed for all chunks and the results are shared with all cohorts.

The client and server components of *sPLINK* has been written using the Python API of the HyFed framework [53]. The WebApp component has been implemented using Angular and HTML/CSS. *sPLINK* employs the algorithm-agnostic compensator of the HyFed framework. The *pandas* package [54] is used in the client component to open the dataset files while *NumPy* [55] is leveraged to pre-process the data and to compute the local parameters. In the server component, the *NumPy* and *SciPy* [56] packages are used for aggregation and computing *p*-values.

## Supplementary Information

**Additional file 1** **Experimental details.Table S1.** The SHIP case study. **Table S2.**. The COPDGene case study. **Table S3.** The FinnGen case study. **Supplementary results.Figure S1.** The significant SNPs overlapped between sPLINK and PLINK for the SHIP case study considering Bonferroni significance threshold. **Figure S2.** The Spearman rank correlation coefficient between the *p*-values from each tool and the aggregated analysis for the COPDGene and FinnGen case studies. **Figure S3.** Runtime and network bandwidth usage of sPLINK with varying number of SNPs. **Figure S4.** Runtime and network bandwidth usage of sPLINK with varying number of samples. **Figure S5.** Runtime and network bandwidth usage of sPLINK with varying number of clients. **Experimental setup.Table S4.** The system specification of the physical machines and laptops used to measure the runtime and network bandwidth usage of sPLINK. **Table S5.** The experimental setup used for measuring the runtime and network bandwidth usage of sPLINK.

**Additional file 2** Review history.

## Data Availability

The SHIP dataset [33] is accessible to researchers after completing a web-based request form at http://ship.community-medicine.de and approval. The COPDGene dataset [35] is publicly available (dbGaP accession number phs000179.v1.p1). The FinnGen dataset [36] is available for researchers by requesting access to the FinnGen Sandbox environment, and after completing Sandbox training on how to deal with personal data, and passing an exam about data security (https://www.finngen.fi/en). The sPLINK tool is available online at https://exbio.wzw.tum.de/splink. The source code of sPLINK is publicly available at GitHub (https://github.com/tum-aimed/splink) and Zenodo (DOI: 10.5281/zenodo.5735472) [57] under the Apache License Version 2.0.

## Footnotes

1 The best result from Kamm et al. [17] has been considered.
