# Supplementary material for: sPLINK: a hybrid federated tool as a robust alternative to meta-analysis in genome-wide association studies
Source: Genome Biol. 2022 Jan 24;23:32. doi: 10.1186/s13059-021-02562-1 (PMC8785575; doi:10.1186/s13059-021-02562-1)
Supplement: Supplementary file 1 — Additional file 1 Experimental details.Table S1. The SHIP case study. Table S2.. The COPDGene case study. Table S3. The FinnGen case study. Supplementary results.Figure S1. The significant SNPs overlapped between sPLINK and PLINK for the SHIP case study considering Bonferroni significance threshold. Figure S2. The Spearman rank correlation coefficient between the p-values from each tool and the aggregated analysis for the COPDGene and FinnGen case studies. Figure S3. Runtime and network bandwidth usage of sPLINK with varying number of SNPs. Figure S4. Runtime and network bandwidth usage of sPLINK with varying number of samples. Figure S5. Runtime and network bandwidth usage of sPLINK with varying number of clients. Experimental setup.Table S4. The system specification of the physical machines and laptops used to measure the runtime and network bandwidth usage of sPLINK. Table S5. The experimental setup used for measuring the runtime and network bandwidth usage of sPLINK. [file 13059_2021_2562_MOESM1_ESM.pdf]

Supplementary material for sPLINK: a hybrid federated tool as a robust alternative to meta-analysis in genome-wide association studies

Reza Nasirigerdeh[1,2], Reihaneh Torkzadehmahani[1], Julian Matschinske[3], Tobias Frisch[4], Markus List[5], Julian Späth[3], Stefan Weiss[6], Uwe Völker[6], Esa Pitkänen[7,8], Dominik Heider[9], Nina Kerstin Wenke[3], Georgios Kaissis[1,2,12,13], Daniel Rueckert[1,2,12], Tim Kacprowski[10,11,*], and Jan Baumbach[3,4,*]

[1]AI in Medicine and Healthcare, Technical University of Munich, Munich, Germany
[2]Klinikum rechts der Isar, Technical University of Munich, Munich, Germany
[3]Chair of Computational Systems Biology, University of Hamburg, Hamburg, Germany
[4]Department of Mathematics and Computer Science, University of Southern Denmark, Odense, Denmark
[5]Chair of Experimental Bioinformatics, TUM School of Life Sciences, Technical University of Munich, Munich, Germany
[6]Department of Functional Genomics, University Medicine Greifswald, Greifswald, Germany
[7]Institute for Molecular Medicine Finland (FIMM), Helsinki Institute of Life Science (HiLIFE), University of Helsinki, Helsinki, Finland
[8]Applied Tumor Genomics Research Program, Research Programs Unit, Faculty of Medicine, University of Helsinki, Helsinki, Finland
[9]Department of Mathematics and Computer Science, University of Marburg, Marburg, Germany
[10]Division Data Science in Biomedicine, Peter L. Reichertz Institute for Medical Informatics of TU Braunschweig and Hannover Medical School, Brunswick, Germany
[11]Braunschweig Integrated Centre of Systems Biology (BRICS), Brunswick, Germany
[12]Biomedical Image Analysis Group, Imperial College London, London, UK
[13]OpenMined, Oxford, UK
[*]Joint last authors

Experimental details

We used *PLINK* V1.9 to generate the splits and perform the aggregated analysis; SNPs with minor allele frequency below 0.05 were filtered out. The common SNPs among the splits have been considered in all analyses. Tables S1-S3 list the sample size (case | control | total) and the number of SNPs for each split in the aggregated analysis with PLINK, meta-analysis using PLINK, METAL, and GWAMA, and the federated analysis using sPLINK.

Table S1 The SHIP case study

**Association test**	**Split1**		**Split2**		**Split3**		**Split4**		**Aggregated**	
	Sample size	# of SNPs	Sample size	# of SNPs	Sample size	# of SNPs	Sample size	# of common SNPs	Sample size	# of common SNPs
Chi-square	229 ∣ 712 ∣ 941	5070067	276 ∣ 768 ∣ 1044	5062964	245 ∣ 761 ∣ 1006	5070192	184 ∣ 524 ∣ 708	5077381	934 ∣ 2765 ∣ 3699	4878280
Logistic regression	229 ∣ 712 ∣ 941	5070067	276 ∣ 768 ∣ 1044	5062964	245 ∣ 761 ∣ 1006	5070192	184 ∣ 524 ∣ 708	5077381	934 ∣ 2765 ∣ 3699	4878280
Linear regression	941	5070067	1044	5062964	1006	5070192	708	5077381	3699	4878280

Table S2 The COPDGene case study

**Scenario**	**Split1**		**Split2**		**Split3**		**Aggregated**	
	Sample size	# of SNPs	Sample size	# of SNPs	Sample size	# of SNPs	Sample size	# of common SNPs
I	937 ∣ 844 ∣ 1781	584910	937 ∣ 844 ∣ 1781	584816	937 ∣ 844 ∣ 1781	585071	2811 ∣ 2532 ∣ 5343	580719
II	737 ∣ 1044 ∣ 1781	584928	937 ∣ 844 ∣ 1781	585108	1137 ∣ 644 ∣ 1781	584816	2811 ∣ 2532 ∣ 5343	580743
III	537 ∣ 1244 ∣ 1781	584978	937 ∣ 844 ∣ 1781	584983	1337 ∣ 444 ∣ 1781	584860	2811 ∣ 2532 ∣ 5343	580783
IV	337 ∣ 1444 ∣ 1781	585105	937 ∣ 844 ∣ 1781	584960	1537 ∣ 244 ∣ 1781	584655	2811 ∣ 2532 ∣ 5343	580709
V	237 ∣ 1544 ∣ 1781	585260	937 ∣ 844 ∣ 1781	585020	1637 ∣ 144 ∣ 1781	584658	2811 ∣ 2532 ∣ 5343	580789
VI	936 ∣ 845 ∣ 1781	585042	936 ∣ 845 ∣ 1781	585073	937 ∣ 844 ∣ 1781	584839	2811 ∣ 2532 ∣ 5343	580719

Table S3 The FinnGen case study

**Scenario**	**Split1**		**Split2**		**Split3**		**Aggregated**	
	Sample size	# of SNPs	Sample size	# of SNPs	Sample size	# of SNPs	Sample size	# of common SNPs
I	22838	997660	22838	997744	22838	997696	68514	994881
II	22838	997751	28547	997962	19983	997604	71368	995016
III	22838	997786	45676	998442	17129	997233	85643	995090
IV	22838	997722	68514	998843	14274	996997	105626	994999
V	22838	997803	99345	999114	12561	996775	134744	994918

Supplementary results

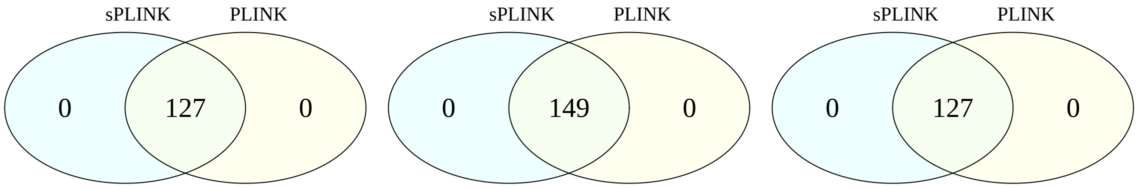

(a) Logistic regression **(b)** Linear regression **(c)** Chi-square test

Fig. S1 The significant SNPs overlapped between *sPLINK* and *PLINK* for the SHIP case study considering **Bonferroni** significance threshold, which is $\approx 1 \times 10^{-8}$ in our case. *sPLINK* and *PLINK* identify the same set of SNPs as significant.

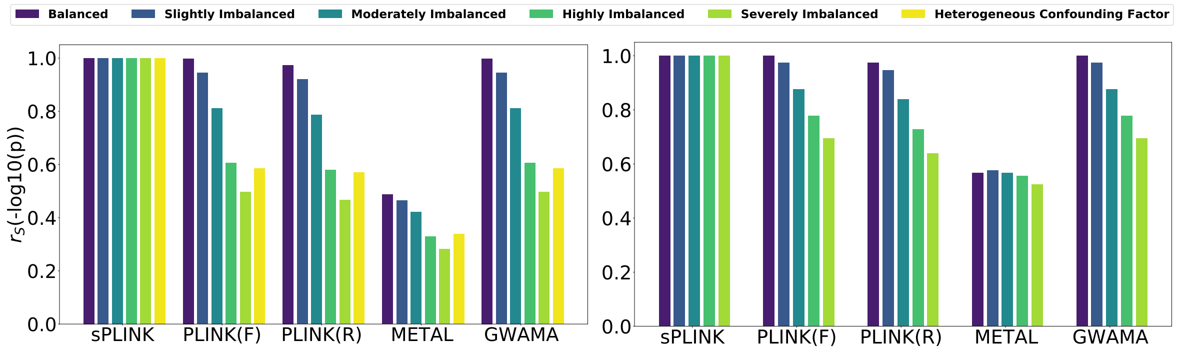

(a) The COPDGene case study **(b)** The FinnGen case study

Fig. S2 The **Spearman** rank correlation coefficient between the p-values from each tool and the aggregated analysis for the COPDGene and FinnGen case studies. *F* and *R* stand for fixed-effect and random-effect, respectively.

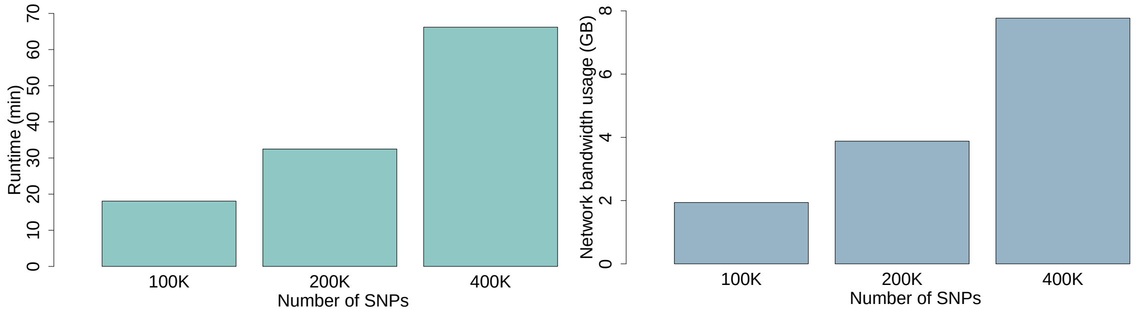

(a) Runtime **(b)** Network bandwidth usage

Fig. S3 Runtime and network bandwidth usage of *sPLINK* with **varying number of SNPs**

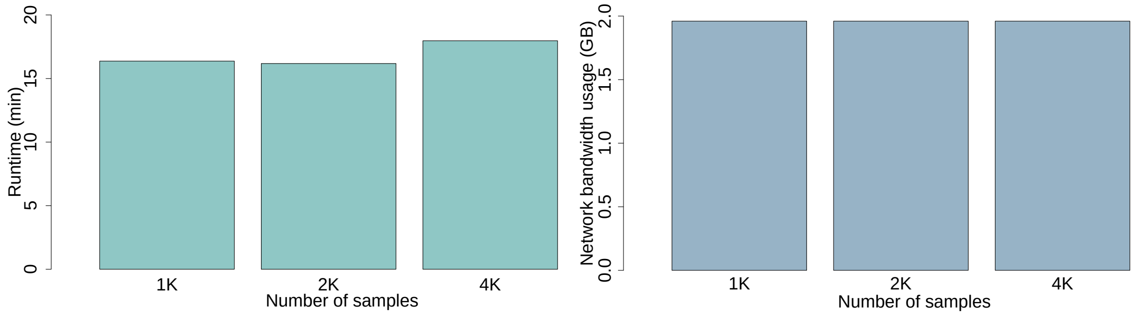

(a) Runtime **(b)** Network bandwidth usage

Fig. S4 Runtime and network bandwidth usage of *sPLINK* with **varying number of samples**

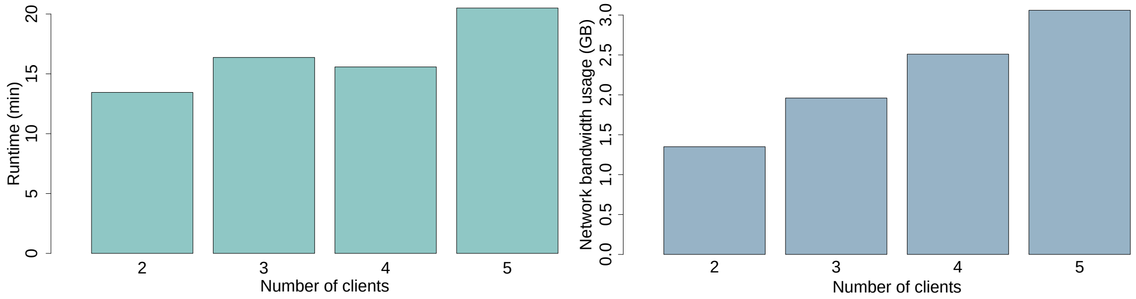

(a) Runtime **(b)** Network bandwidth usage

Fig. S5 Runtime and network bandwidth usage of *sPLINK* with **varying number of clients**

Experimental setup

Table S4 The system specification of the physical machines and laptops used to measure the runtime and network bandwidth usage of sPLINK; Download/upload speeds are approximate values measured using *speedtest-cli* (`https://github.com/sivel/speedtest-cli`); GB: Gigabyte; Mbps: Megabit per second

System name	# of cores used	Memory size (GB)	Upload (Mbps)	Download (Mbps)	Location	Experiment sets used
Server	8	12	411	527	Freising	All
Compensator	4	12	810	830	Odense	All
Laptop1	4	16	35	76	Munich	All
Laptop2	4	16	10	58	Freising	All
Laptop3	4	8	24	21	Freising	1
Laptop4	4	8	95	93	Freising	4
Desktop-PC	4	64	11	93	Freising	2,3,4

Table S5 The experimental setup used for measuring the runtime and network bandwidth usage of sPLINK; COPDGene is employed as the dataset in all experiment sets; logistic regression is used in experiment sets 2-4; In the first experiment of the experiment set 2 (i.e. sample size 1781 and SNP count 100K), 12 cores of the Desktop-PC system is used instead of 4; K: 1000

Experiment set #	Description	# of clients	Sample size per client	# of SNPs	Chunk size	Beta iterations
1	chi-square | linear | logistic	3	1781	$\sim 580K$	200K	- | - | 20
2	varying # of SNPs	3	1781	100K, 200K, 400K	100K	20
3	varying # of samples	3	1K, 2K, 4K	100K	100K	5
4	varying # of clients	2,3,4,5	1K	100K	100K	5
