# Supplementary material for: sPLINK: a hybrid federated tool as a robust alternative to meta-analysis in genome-wide association studies
Source: Genome Biol. 2022 Jan 24;23:32. doi: 10.1186/s13059-021-02562-1 (PMC8785575; doi:10.1186/s13059-021-02562-1)
Supplement: Supplementary file 2 — Additional file 2 Review history. [file 13059_2021_2562_MOESM2_ESM.docx]

Review History

**First round of review**

**Reviewer 1**

**Were you able to assess all statistics in the manuscript, including the appropriateness of statistical tests used?**

Yes, and I have assessed the statistics in my report.

**Were you able to directly test the methods?**

No

**Comments to author:**

The authors introduce sPLINK, a federated tool for genome-wide-association studies. When data sharing across different institutions is challenging due to regulatory constraints, meta-analysis approaches are typically used to conduct GWAS on the combined data based on the aggregate statistics computed on each dataset. The authors show that meta-analysis suffers from reduction in power due to cross-study heterogeneity and proposes a federated analysis pipeline that replicates the results of a pooled analysis without sharing individual-level data. They evaluate this approach on three existing GWAS datasets with simulated partitions. Software clients for sPLINK are provided along with a web interface. The authors address a key challenge in the field, and the results showing the effects of cross-study heterogeneity are informative. However, technical innovation appears limited as distributed analysis approaches for linear/logistic regression have been well-studied (as the authors have noted). The privacy-preserving aspect of sPLINK is questionable, given a lack of discussion on the safety of sharing intermediate results. In addition, the issue of population stratification has not been addressed, which is an important confounding factor in GWAS, especially in multi-cohort settings.

Major comments

- From a technical standpoint, I found the manuscript's characterization of the improvement of sPLINK over the prior art to be misleading. sPLINK closely resembles a previous tool called GLORE [1], which the authors have cited. Both perform gradient iterations to estimate the coefficients of logistic regression models. It is explained in Table 2 that sPLINK is an improvement over GLORE because it is robust to heterogeneity and additionally implements linear regression. However, GLORE is fundamentally based on the same model as sPLINK and directly extends to the setting with confounding factors in the same manner as sPLINK. On the other hand, distributed linear regression approach of sPLINK is a known approach dating back to 2005 [2]. Providing easy-to-use software implementations of existing techniques is still a valuable contribution; however, these contexts need to be more clearly communicated in the manuscript. Part of the issue seems to be that all of the descriptions regarding the techniques used in sPLINK are deferred to the supplement and not presented clearly in the main text. In addition, when describing existing distributed analysis approaches based on HE and SMPC, a clear distinction needs to be made that these cryptographic solutions provide privacy guarantees that are fundamentally stronger than federated learning.

- The paper focuses on matching the output of GWAS with the vanilla GWAS workflows provided in PLINK, and although this is meaningful, it does not address the most common use case of GWAS for heterogeneous study populations that require correction for population stratification via methods such as PCA and linear mixed models (LMM). This is especially important given the paper's emphasis on cross-study heterogeneity, a large part of which will likely be due to ancestry differences in the cohorts that are not explicitly codified as covariate features in the datasets. Rui et al. [4] introduced a federated analysis approach to generalized LMM for GWAS analysis, and GWAS based on PCA has also been previously proposed by Cho et al. [3]. Without the support for these components, applicability of sPLINK is likely to be limited.

- Federated learning does not guarantee privacy. A noticeable gap in the presentation of sPLINK is the assumption that not sharing individual-level data is enough for privacy protection. Without a careful explanation of what kinds of intermediate results are being shared and why it is safe to do so, claiming that sPLINK is privacy-preserving would be misleading, especially given the existence of other proposals based on HE/SMPC with strong privacy properties.

[1] https://doi.org/10.1136/amiajnl-2012-000862

[2] https://doi.org/10.1198/106186005X47714

[3] https://doi.org/10.1038/nbt.4108

[4] https://doi.org/10.1093/bioinformatics/btaa478

Minor comments

- Figure 3a-c show a peculiar error pattern where the sPLINK results deviate from PLINK in specific genomic positions. The authors attribute this to floating point precision error, but this does not seem compelling given the highly targeted nature of the error. Further investigation seems needed.

- Figures 3d-f show overlap in GWAS hits between PLINK and sPLINK but for a fixed p-value threshold. It would be more informative to present the overlap across a range of thresholds

- "Identified SNPs have also been implicated in a pervious analysis of this dataset": No information is given about what these identified SNPs are.

- As shown in Fig 6, meta-analysis approaches miss many of the significant SNPs due to confounding. Are the p-values generally deflated due to reduced power, but otherwise maintain accurate ordering of significance compared to pooled studies?

**Reviewer 2**

**Were you able to assess all statistics in the manuscript, including the appropriateness of statistical tests used?**

Yes, and I have assessed the statistics in my report.

**Comments to author:**

In this paper, the author applied a federated learning scheme to perform GWAS on distributed datasets by preserving the privacy of datasets. Performing linear (or logistic) regression on federated datasets has been studied in the field of cryptography. In this paper the authors focused on the application of these existing methods in GWAS.

Main comments:

- The authors should provide the clear differences between the proposed method with previous works on GWAS in federated dataset (Scot et al, 2015, 10.1186/1472-6947-15-S5-S2 , 10.1109/TCBB.2018.2829760)

- A major computational bottleneck for secure GWAS is correcting for population structure, which can cause spurious associations that reflect interpopulation differences, rather than a true biological signal (Freedman, M.L. et al 2004). A widely used procedure is to include the top principal component analysis (PCA) as covariates which are thought to be representative of population-level differences among individuals as in the subsequent association tests to correct for bias. The authors did not discuss this issue and how they want to tackle this problem. Here is a paper (https://www.nature.com/articles/nbt.4108) proposing a secure way to compute PCs and use them in GWAS using multiparty computation. There should be a way to compute PCs in federated dataset as well.

- There are some key parameters required for practical GWAS such as HWE, MAF and LD. The authors did not talk about how they compute those parameters securely in their framework.

- Figure 2 shows the difference between proposed method and meta-Analysis without providing any details about the experiment (sample sizes, number of SNPs, ...).

- Figure 3 in SI which is about running time must be moved to the main text. The authors should provide the computational efficiency of their method by varying the different parameters such as number of samples, number of SNPs, number of databases.

**Authors Response**

**Point-by-point responses to the reviewers’ comments:**

Reviewer #1

The authors introduce sPLINK, a federated tool for genome-wide-association studies. When data sharing across different institutions is challenging due to regulatory constraints, meta-analysis approaches are typically used to conduct GWAS on the combined data based on the aggregate statistics computed on each dataset. The authors show that meta-analysis suffers from reduction in power due to cross-study heterogeneity and proposes a federated analysis pipeline that replicates the results of a pooled analysis without sharing individual-level data. They evaluate this approach on three existing GWAS datasets with simulated partitions. Software clients for sPLINK are provided along with a web interface. The authors address a key challenge in the field, and the results showing the effects of cross-study heterogeneity are informative. However, technical innovation appears limited as distributed analysis approaches for linear/logistic regression have been well-studied (as the authors have noted). The privacy-preserving aspect of sPLINK is questionable, given a lack of discussion on the safety of sharing intermediate results. In addition, the issue of population stratification has not been addressed, which is an important confounding factor in GWAS, especially in multi-cohort settings.

Major comment #1: From a technical standpoint, I found the manuscript's characterization of the improvement of sPLINK over the prior art to be misleading. sPLINK closely resembles a previous tool called GLORE [1], which the authors have cited. Both perform gradient iterations to estimate the coefficients of logistic regression models. It is explained in Table 2 that sPLINK is an improvement over GLORE because it is robust to heterogeneity and additionally implements linear regression. However, GLORE is fundamentally based on the same model as sPLINK and directly extends to the setting with confounding factors in the same manner as sPLINK. On the other hand, distributed linear regression approach of sPLINK is a known approach dating back to 2005 [2]. Providing easy-to-use software implementations of existing techniques is still a valuable contribution; however, these contexts need to be more clearly communicated in the manuscript. Part of the issue seems to be that all of the descriptions regarding the techniques used in sPLINK are deferred to the supplement and not presented clearly in the main text. In addition, when describing existing distributed analysis approaches based on HE and SMPC, a clear distinction needs to be made that these cryptographic solutions provide privacy guarantees that are fundamentally stronger than federated learning.

*We revised the paragraphs corresponding to the federated tools for GWAS and their comparison with sPLINK in the “Background” section to clarify the novelty and advantages of sPLINK as follows:*

*“To address the computational limitation of HE/SMPC-based methods, the association tests can be implemented in a federated fashion. Federated learning-based methods follow the paradigm of "move computation to data", distributing the heavy computations among the cohorts while performing lightweight aggregation (simple operations such as addition and multiplication of the parameters) at the central server. Wang et al. introduced EXPLORER for distributed and privacy-preserving logistic regression algorithm. EXPLORER is a model but not a tool for GWAS. Moreover, it does not provide a “guarantee for optimal global solution”, implying that its results can be different from the aggregated analysis in general. GLORE implemented a federated logistic regression test for GWAS. In GLORE, the parameter values computed by the cohorts are revealed to the server. Studies showed that in general, this practice can lead to privacy leakage, where the data of the cohorts might be reconstructed by the server. Moreover, even if two federated algorithms such as principal component analysis (PCA) and linear regression are individually privacy-preserving, their combination in a pipeline, where the output of the former is employed as part of the input for the latter (population stratification in GWAS), is not necessarily privacy-preserving if the server has the real values of the parameters.”*

*“Several hybrid federated frameworks including HyFed have been introduced to deal with the privacy concerns of federated learning. HyFed combines federated learning with a quasi-SMPC approach to hide the original values of the local parameters from the other parties including the server. It is a suitable framework for developing federated GWAS algorithms because it provides enhanced privacy while preserving the accuracy of the results. It also supports federated mode, where different components can run in separate physical machines and securely communicate with each other over the Internet.”*

*“In this paper, we present a novel tool set called sPLINK (safe PLINK) based on the HyFed framework for federated and privacy-preserving GWAS. sPLINK consists of four main components (Figure 2): Web application (WebApp) to configure the parameters (e.g. association test) of the new study; client to compute the local parameters from the data, perturb them with noise, and share the noise with compensator and noisy local parameters with server; compensator to aggregate the noise values of the clients and send the aggregated noise to the server; server to compute the global parameters by adding up the noisy local parameters and the negative of the aggregated noise. Notice that the utility of the global model is preserved because the aggregated noise from the compensator cancels out the accumulated noise from the noisy local parameters during the aggregation. Unlike PLINK, sPLINK is applicable to distributed data while protecting privacy. In sPLINK, neither the private data of cohorts leaves the site nor the real values of the local parameters are revealed to the other parties (Figure 1c). Contrary to existing HE/SMPC-based methods,sPLINK is computationally efficient since heavy computations are distributed across the cohorts while simple aggregation is performed on the server and compensator. Compared to the current federated GWAS tools like GLORE, sPLINK not only provides enhanced privacy but also supports multiple association tests including logistic regression, linear regression, and chi-square for GWAS.”*

*Additionally, we updated Table2 in the “Results” section to clarify that GLORE is not privacy-preserving in general because the clients reveal the real values of the model parameters to the server.*

*We also added the following to the “Discussion” section as a brief comparison between sPLINK and HE/SMPC-based approaches from the privacy perspective:*

*“Notice that the HE-based approaches offer stronger privacy than sPLINK but with more computational overhead and less accurate results. sPLINK provides stronger privacy compared to the SMPC-based approaches because if parties collude in sPLINK, only the model parameters are revealed whereas the private data is leaked if collusion occurs in the SMPC-based tools.”*

Major comment #3: Federated learning does not guarantee privacy. A noticeable gap in the presentation of sPLINK is the assumption that not sharing individual-level data is enough for privacy protection. Without a careful explanation of what kinds of intermediate results are being shared and why it is safe to do so, claiming that sPLINK is privacy-preserving would be misleading, especially given the existence of other proposals based on HE/SMPC with strong privacy properties.

*To address this concern, we re-implemented sPLINK using the HyFed framework to hide the real values of the model parameters from the server, and updated the manuscript according to the new architecture and implementation.*

Minor comment #1: Figure 3a-c show a peculiar error pattern where the sPLINK results deviate from PLINK in specific genomic positions. The authors attribute this to floating point precision error, but this does not seem compelling given the highly targeted nature of the error. Further investigation seems needed.

*The vertical axis of Figure 3a-c indicates ∆log10(p)= log10(p2) - log10(p1), where p1 and p2 are p-values from the aggregated analysis and sPLINK, respectively. We use ∆log10(p) instead of ∆p = (p2 - p1) because it is a better indicator of the difference between two small p-values. As we mentioned in the paper “the maximum difference is 0.162 for a SNP in the linear regression. sPLINK and PLINK report 4.441 × 10−16 and 3.058 × 10−16 as p-values for the SNP, respectively.” The difference between the p-values is NOT 0.162 but it is: ∆p = 4.441 × 10−16 - 3.058 × 10−16 = 1,383 × 10−16 , which is negligible. We also double checked the results for the SNPs from chromosome #9, where we have high ∆log10(p). There are dozens of significant SNPs in chromosome #9, whose p-values are around 10−16 . While ∆log10(p) is in the order of 0.1 or 0.01, the difference between the p-values reported by sPLINK and PLINK is negligible (in order of 10−16).*

Minor comment #2: Figures 3d-f show overlap in GWAS hits between PLINK and sPLINK but for a fixed p-value threshold. It would be more informative to present the overlap across a range of thresholds.

*We also considered the Bonferroni significance threshold (in our case, it was 1.0530137675232022e-08) to compare the overlapped significant SNPs in PLINK and sPLINK. The results remained similar and we added the Figure to Supplementary B. We also added the following sentences in the manuscript:*

*“We leverage the Bonferroni significance threshold (which is ≈ 1×10−8 for our tests) to compare the overlapping significant SPNs from sPLINK and PLINK. The results remain similar and the associated plot (Figure 9) is available at Supplementary B.”*

Minor comment #3: "Identified SNPs have also been implicated in a previous analysis of this dataset": No information is given about what these identified SNPs are.

*We have updated the manuscript and included the information about the SNP’s based on the referenced paper. For further biological details we kindly refer to the cited paper.*

Minor comment #4: As shown in Fig 6, meta-analysis approaches miss many of the significant SNPs due to confounding. Are the p-values generally deflated due to reduced power, but otherwise maintain accurate ordering of significance compared to pooled studies?

*We calculated the Spearman rank correlation for Figure 6 to check whether or not the meta-analysis tools maintain the ordering of significance compared with the aggregated analysis. Our results showed that: (1) the Spearman correlation values for the meta-analysis tools are lower in general compared to the Pearson correlation values, and (2) as we increase the heterogeneity of the phenotypes across splits, the Spearman correlation reduces for the meta-analysis tools, similar to the behavior we see if we employ the Pearson correlation. We added the plot to Supplementary B and the following paragraph to the manuscript:*

*“We also leverage the Spearman correlation to check whether or not the meta-analysis tools maintain the ordering of significance compared to the aggregated analysis. Our results show that this is not the case, and the Spearman correlation values for the meta-analysis tools reduce as the phenotype imbalance across the splits increases, similar to the results from Figure 6, where the Pearson correlation is used. The corresponding figure (Figure 10) can be found in Supplementary B.”*

Reviewer #2

In this paper, the author applied a federated learning scheme to perform GWAS on distributed datasets by preserving the privacy of datasets. Performing linear (or logistic) regression on federated datasets has been studied in the field of cryptography. In this paper the authors focused on the application of these existing methods in GWAS.

Major comment #1: The authors should provide the clear differences between the proposed method with previous works on GWAS in federated dataset (Scot et al, 2015, 10.1186/1472-6947-15-S5-S2 , 10.1109/TCBB.2018.2829760)

*We mentioned the SMPC-based method by Scott Constable et alas well as the SAFETY framework by Nazmus Sadat et al. in the “Background” section as follows:*

*“Kamm et al and Cho et al. proposed secure GWAS frameworks based on SMPC. The former developed simple association tests including Cochran–Armitage and chi-square (χ2) and the latter implemented only Cochran–Armitage test. Shi et al. presented a secure SMPC-based logistic regression framework for GWAS. Constable et al. implemented an SMPC-based framework for minor allele frequency and chi-square computation. These frameworks inherit the limitations of SMPC itself: They follow the paradigm of "move data to computation", where they put the processing burden on a few computing parties. Consequently, they are computationally expensive and are not scalable for large-scale GWAS. Moreover, they suffer from the colluding-parties problem in which, if the parties send the secret shares of the cohorts to each other, the whole private data of the cohorts is exposed.”*

*“Lu et al., Morshed et al., and Kim et al. developed secure chi-square test, linear regression, and logistic regression using HE for GWAS, respectively. Sadat et al. introduced the SAFETY framework based on HE and Intel Software Guard Extensions technology, which implements the Linkage Disequilibrium, Fisher’s Exact Test, Cochran-Armitage Test for Trend, and Hardy-Weinberg Equilibrium statistical tests. Similar to SMPC-based methods, they are not computationally efficient since a single server carries out operations over encrypted data,causing considerable overhead26. Additionally, HE-based methods introduce accuracy loss in the association test results. This is because HE only supports addition and multiplication, and as a result, non-linear operations in regression tests should be approximated using those two operations.”*

Major comment #3: There are some key parameters required for practical GWAS such as HWE, MAF and LD. The authors did not talk about how they compute those parameters securely in their framework.

*We added the following to the “Discussion” section to address that:*

*“sPLINK currently supports chi-square and linear/logistic regression tests, but it can be extended to compute other useful statistics in GWAS such as minor allele frequency (MAF), Hardy-Weinberg equilibrium (HWE), and linkage disequilibrium (LD) between SNPs in a privacy-preserving manner. The federated computation of the aforementioned statistics in sPLINK is expected to be straightforward because they are based on the allele frequencies, and sPLINK already calculates the minor and major allele counts in the Non-missing count step of its computational workflow (Section 5).”*

Major comment #4: Figure 2 shows the difference between proposed method and meta-Analysis without providing any details about the experiment (sample sizes, number of SNPs, ...).

*We added the details of the experiments to Supplementary A.*

Major comment #5: Figure 3 in SI which is about running time must be moved to the main text. The authors should provide the computational efficiency of their method by varying the different parameters such as number of samples, number of SNPs, number of databases.

*We moved the runtime and network bandwidth usage results for sPLINK to the main text in the “Results” section. We also measured the aforementioned metrics with varying number of SNPs, samples, and clients and provided the plots in Supplementary B but discussed them in the “Results” section:*

*“Figure 7a plots the sPLINK’s runtime for each association test. sPLINK computes the results for chi-square, linear regression, and logistic regression in 8 min, 20 min, and 75 min, respectively. Sending parameters from the clients to the server and compensator contributes the most in sPLINK’s runtime. Compared to Kamm et al., sPLINK is almost 13 times faster for chi-square test (8 min vs. 110 min) with less powerful hardware, larger sample size (5343 vs. 1080), and more number of SNPs (- 580K vs. - 263K). In contrast to Cho et al., sPLINK is faster by a factor of -8 (1.25h vs. - 10.3h) comparing logistic regression test from sPLINK to Cochran–Armitage test implemented by that study. Notice that logistic regression is more complex algorithm than Cochran–Armitage.”*

*“Figure 7b depicts the network usage of sPLINK. The clients, server, and compensator exchange total of 0.967 GB, 2.49 GB, and 11.06 GB traffic in chi-square, linear regression, and logistic regression, respectively. Logistic regression has higher volume of traffic exchange since the computation of beta coefficients are performed in an iterative fashion. A fair comparison between sPLINK and SMPC-based frameworks from the network communication aspect is tricky. However, in general, (hybrid) federated learning-based approaches consume more network bandwidth than SMPC-based ones.“*

*“We also conducted a set of experiments to investigate how the runtime and network bandwidth consumption of sPLINK change with varying number of samples, SNPs, and clients. The results demonstrate that the traffic exchanged over the network is independent of the sample size and linearly increases with the number of SNPs and clients (as expected). Moreover, runtime is not affected much by the sample size thanks to the multi-threading capability of sPLINK’s client package, and linearly/non-linearly increases with the number of SNPs/clients. The corresponding plots (Figures 11-13) are available in Supplementary B.”*

Major comment #2 of Reviewer #1: The paper focuses on matching the output of GWAS with the vanilla GWAS workflows provided in PLINK, and although this is meaningful, it does not address the most common use case of GWAS for heterogeneous study populations that require correction for population stratification via methods such as PCA and linear mixed models (LMM). This is especially important given the paper's emphasis on cross-study heterogeneity, a large part of which will likely be due to ancestry differences in the cohorts that are not explicitly codified as covariate features in the datasets. Rui et al. [4] introduced a federated analysis approach to generalized LMM for GWAS analysis, and GWAS based on PCA has also been previously proposed by Cho et al. [3]. Without the support for these components, applicability of sPLINK is likely to be limited.

Major comment #2 of Reviewer #2: A major computational bottleneck for secure GWAS is correcting for population structure, which can cause spurious associations that reflect interpopulation differences, rather than a true biological signal (Freedman, M.L. et al 2004). A widely used procedure is to include the top principal component analysis (PCA) as covariates which are thought to be representative of population-level differences among individuals as in the subsequent association tests to correct for bias. The authors did not discuss this issue and how they want to tackle this problem. Here is a paper (https://www.nature.com/articles/nbt.4108) proposing a secure way to compute PCs and use them in GWAS using multiparty computation. There should be a way to compute PCs in federated dataset as well.

*We plan to add population stratification to sPLINK in a future version since this extension is not straightforward. We discussed the challenges and general ideas in the “Discussion” section as follows:*

*“Moreover, population stratification using PCA will be addressed in the future version of sPLINK due to the complexity of the problem. sPLINK’s implementation of the association tests is horizontally-federated, where the datasets have different samples but the same features (i.e. SNP and confounding factors). However, correcting for population structure using sPLINK requires a vertically-federated PCA algorithm because the eigenvectors should be computed from the sample by sample covariance matrix, and therefore, the samples and features swap roles in the federated PCA (SNPs are considered as samples and patients as features). Vertical federated learning algorithms are still understudied, and they are considered more complicated than the horizontal algorithms. “*

*“Additionally, the federated PCA algorithm should be an iterative, randomized algorithm so that it can handle large GWAS datasets with a practical amount of memory. The iterative nature of the algorithm will present network and runtime challenges because it might need dozens or hundreds of iterations and exchange huge traffic over the network to converge to final eigenvectors. From the privacy perspective, a recent study demonstrates that even if a federated PCA algorithm and linear regression are individually privacy-preserving, federated population stratification in GWAS, where the eigenvectors are used as the confounding factors in the association test, is not necessarily privacy-preserving. Consequently, the server can reconstruct the SNP or binary confounding factor values in polynomial time. To tackle this issue, they suggested that the final eigenvectors should be computed at the clients and the model parameter values should be hidden from the server. The federated population stratification in sPLINK should be implemented taking into account those suggestions.”*

**Second round of review**

**Reviewer 1**

The revised sPLINK now uses HyFed framework for federated learning, which is a recently proposed method by the same authors (not yet published). This is meant to address the prior review comment that federated learning is not necessarily private.

Throughout the manuscript, it is implied that the use of HyFed resolves the privacy concerns related to federated learning solutions. While privacy is indeed improved compared to the original sPLINK, a comprehensive discussion of the remaining risks and potential threats are again omitted from the manuscript. HyFed does not protect leakage in the aggregated intermediate data, which are revealed (denoised) and made visible to the coordinator server and each cohort. Just as the local intermediate data can reveal sensitive info, the aggregated data can also reveal sensitive info, albeit at the level of the pooled dataset. Note that if the outcome is the reconstruction of an individual’s genotype data, then whether or not this was done at the pooled level or cohort level is not too important. This is a smaller but still significant risk considering the iterative nature of the aggregation steps -- especially if the cohorts are small. These issues are not discussed and mitigated for in the manuscript.

The fact that HyFed relies on a “quasi” SMPC approach for secure aggregation of intermediate data, even though there are more rigorous and established approaches for this purpose (e.g. [1]) is another source of concern. The authors follow the high-level intuition of secret sharing (core technique in SMPC) by splitting the intermediate data into two parts (data+noise and the noise itself) and upload them individually to two geographically separated parties. This hides the data shared by each party while allowing the data to be aggregated across parties. However the technical details of HyFed diverge from standard secret sharing frameworks and come across as being ad hoc; why haven’t the authors used (or compared to) existing secure aggregation methods? No formal statement and proof of security are provided for the authors’ technique which are needed to build trust in their tools. I also could not find these justifications in the HyFed preprint. Unfortunately, I don’t think this meets the standard for a privacy-focused publication.

[1] https://doi.org/10.1145/3133956.3133982

The authors have chosen not to address the population stratification problem in this manuscript. In this case the claim that sPLINK is robust to heterogeneity needs to be clearly qualified in scope because population heterogeneity across cohorts is likely a major source of heterogeneity that sPLINK is not able to address, unlike aggregated analyses.

Minor comments

In Figure 1c, the proposed approach is depicted as procedurally equivalent to meta-analysis, where aggregate statistics are shared among collaborators and in turn the final results are computed based on those statistics. The fact that sPLINK relies on iterative sharing of aggregate statistics is not conveyed.

The way the authors describe their masking procedure as “perturbation with noise” can be confused with differential privacy mechanisms. I recommend “blinding” or “masking” for clarity.

“In contrast to Cho et al., sPLINK is faster by a factor of ∼8 (1.25h vs. ∼ 10.3h)”: Cho et al.’s method includes PCA. Is this a fair comparison? Also, I believe their Armitage trend test is equivalent to linear regression. Table 2 needs to be updated.

Figure 3a-c: I suggest explaining in the caption that the spike in noise in a specific region is due to strong association signals resulting in higher absolute error. Otherwise, these figures give the impression of statistical anomaly.

I disagree with the statement that HyFed is more secure than SMPC. In the event of collusion this is true, but when the respective security assumptions are held as intended, HyFed leaks poorly-understood amount of information through aggregated intermediate data whereas SMPC maintains security throughout the analysis.

**Reviewer 2**

The authors have addressed my major concerns.

**Authors Response**

**Point-by-point responses to the reviewers’ comments:**

Reviewer #1

Major comment #1: The revised sPLINK now uses HyFed framework for federated learning, which is a recently proposed method by the same authors (not yet published). This is meant to address the prior review comment that federated learning is not necessarily private.

Throughout the manuscript, it is implied that the use of HyFed resolves the privacy concerns related to federated learning solutions. While privacy is indeed improved compared to the original sPLINK, a comprehensive discussion of the remaining risks and potential threats are again omitted from the manuscript. HyFed does not protect leakage in the aggregated intermediate data, which are revealed (denoised) and made visible to the coordinator server and each cohort. Just as the local intermediate data can reveal sensitive info, the aggregated data can also reveal sensitive info, albeit at the level of the pooled dataset. Note that if the outcome is the reconstruction of an individual’s genotype data, then whether or not this was done at the pooled level or cohort level is not too important. This is a smaller but still significant risk considering the iterative nature of the aggregation steps -- especially if the cohorts are small. These issues are not discussed and mitigated for in the manuscript.

The fact that HyFed relies on a “quasi” SMPC approach for secure aggregation of intermediate data, even though there are more rigorous and established approaches for this purpose (e.g. [1]) is another source of concern. The authors follow the high-level intuition of secret sharing (core technique in SMPC) by splitting the intermediate data into two parts (data+noise and the noise itself) and upload them individually to two geographically separated parties. This hides the data shared by each party while allowing the data to be aggregated across parties. However the technical details of HyFed diverge from standard secret sharing frameworks and come across as being ad hoc; why haven’t the authors used (or compared to) existing secure aggregation methods? No formal statement and proof of security are provided for the authors’ technique which are needed to build trust in their tools. I also could not find these justifications in the HyFed preprint. Unfortunately, I don’t think this meets the standard for a privacy-focused publication.

*We agree with the reviewer that our approach does not offer a privacy guarantee and that some risks remain. To better reflect this, we now distinguish between the “privacy-aware” and “privacy-preserving” definitions and discuss the limitations of the privacy-aware solutions. We expanded the discussion as follows:*

*“In a privacy-aware approach, it is not required to share the private data with a third party. A privacy-aware approach is privacy-preserving if the approach offers a privacy guarantee that captures the privacy risk associated with individual samples in the dataset.”*

*“To our knowledge, differential privacy (DP) and DP-based hybrid federated learning can offer such a guarantee at the cost of the utility of the model and are considered as privacy-preserving approaches.”*

*“Meta-analysis, SMPC, HE, federated learning, and hybrid federated learning based on SMPC are privacy-aware because they share knowledge (e.g. model) from the data but not the data itself with a third party. In meta-analysis/federated learning, the summary statistics/model parameters of each cohort are shared with a third party. In SMPC, each computing party sends its intermediate results to the other parties. In SMPC-based hybrid federated learning, the aggregated (global) parameters are revealed to the server and cohorts. These approaches, including HE, reveal the final model too. However, these methods are not privacy-preserving because none of them provides a privacy guarantee indicating to what extent the revealed knowledge leaks the private information about a particular sample of the dataset.”*

*“While privacy-aware approaches do not offer a privacy guarantee, they might provide stronger/weaker privacy compared to each other based on the amount and nature of the information they share with third parties. For instance, HE-based methods provide stronger privacy because they only reveal the final model (results) while the other privacy-aware approaches disclose not only the final results but also other information such as summarystatistics or local parameters. Similarly, sPLINK provides enhanced privacy in comparison with existing federated learning based tools such as GLORE. This is because GLORE discloses the local parameters of each cohort to the server, which is not revealed in sPLINK.”*

*We agree with the reviewer that in sPLINK (and HyFed), the aggregated parameters and final results are revealed to the server and cohorts. However, this is also the case for other SMPC-based hybrid federated learning methods including the study [1] mentioned by the reviewer. Here is the related discussion in the manuscript from the Google researchers [2]:*

*“First of all, to prevent the participating nodes from exploiting individually submitted model updates, existing secure aggregation protocols could be used. A practical secure aggregation protocol already used in cross-device FL was proposed by Bonawitz et al. [80], effectively handling dropping out participants at the cost of complexity of the protocol.”*

*“In order to prevent any client from trying to reconstruct the private data of another client by exploiting the global model, client-level differential privacy [338] has been proposed for FL.”*

*Notice that all privacy-aware methods including meta-analysis, the gold standard in GWAS, reveal some information to the third parties but none of them provides a privacy guarantee that revealing such information never leads to privacy leakage or to what extent disclosing such information leaks the privacy of samples in the dataset. We emphasize that such a privacy guarantee is possible with differential privacy but the utility (accuracy) of the results are highly affected in that case.*

*Regarding the question “why haven’t the authors used (or compared to) existing secure aggregation methods?”, we note that the authors of the referenced study [1] have not made their source code publicly available. Moreover, the proposed method suffers from the same limitation as it still reveals the aggregated parameters like HyFed. The system proposed in [1] introduces additional complexity aimed specifically at handling dishonest parties which might not follow the protocol. In sPLINK, we assume honest-but-curious parties.*

*The reviewer also states: “Note that if the outcome is the reconstruction of an individual’s genotype data, then whether or not this was done at the pooled level or cohort level is not too important.” We believe that hiding the local parameters of each client, as [1] and HyFed do, is a significant privacy improvement although it does not address all the privacy concerns. Previous studies including studies [1] (Google) and [4] (IBM) are clear examples for the importance of this research direction.*

*With regard to the comment “No formal statement and proof of security are provided for the authors’ technique which are needed to build trust in their tools. I also could not find these justifications in the HyFed preprint.”, HyFed employs an additive secret sharing scheme in which the noise is one secret share and the noisy model is another secret share; Server and compensator can be considered as the computing parties of the SMPC. The only deviation from the standard SMPC is that only one computing party (compensator) sends the intermediate results to the other party instead of exchanging the intermediate results between two parties. The proof that using this scheme does not reveal the individual local parameters of the clients under the assumptions we already mentioned in the manuscript (honest-but-curious parties that follow the protocol but try to reconstruct the data from the available information, no collusion, and at least two clients) can be found in [3]. Here is a portion of the abstract from [3]: “In this paper we show how to divide data D into n pieces in such a way that D is easily reconstructable from any k pieces, but even complete knowledge of k - 1 pieces reveals absolutely no information about D”*

*Responding to the comment “ While privacy is indeed improved compared to the original sPLINK, a comprehensive discussion of the remaining risks and potential threats are again omitted from the manuscript” and in light of everything discussed above, we have expanded the “Discussion” section to address this.*

*“Notice that although sPLINK significantly enhances the privacy of data compared to existing federated learning tools, it does not eliminate the possibility of data reconstruction using the aggregated parameters or final results. For example, the XTX parameter (covariance matrix) in the linear regression algorithm can be exploited to determine the sex of the patients if the total number of samples across all cohorts is comparable to the number of the confounding factors. However, for a reliable GWAS study, the total sample size is considerably larger than the number of confounding factors, and therefore, the reconstruction of the cohorts’ private data from the aggregated parameters can be difficult (but still possible) in practice. A similar argument is also applicable to meta-analysis approaches, which reveal the summary statistics of each cohort to a third party.”*

*[1]	https://doi.org/10.1145/3133956.3133982*

*[2]	https://arxiv.org/abs/1912.04977*

*[3]	https://dl.acm.org/doi/abs/10.1145/359168.359176*

*[0]	https://dl.acm.org/doi/abs/10.1145/3338501.3357370*

Major comment #2: The authors have chosen not to address the population stratification problem in this manuscript. In this case the claim that sPLINK is robust to heterogeneity needs to be clearly qualified in scope because population heterogeneity across cohorts is likely a major source of heterogeneity that sPLINK is not able to address, unlike aggregated analyses.

*We added a paragraph to the discussion section to clarify this topic:*

*“We showed that sPLINK is robust against an important source of data heterogeneity, namely the heterogeneous distribution of the phenotype or confounding factor values across the distributed datasets of the cohorts. Population heterogeneity across the cohorts is another source of data heterogeneity in GWAS, which is commonly tackled by population stratification using the PCA algorithm. sPLINK currently does not address this kind of data heterogeneity but the future versions of the tool will support population stratification to this end. ”*

Minor comment #1: In Figure 1c, the proposed approach is depicted as procedurally equivalent to meta-analysis, where aggregate statistics are shared among collaborators and in turn the final results are computed based on those statistics. The fact that sPLINK relies on iterative sharing of aggregate statistics is not conveyed.

*We updated Figure1c and the caption to take into account the iterative nature of sPLINK .*

Minor comment #2: The way the authors describe their masking procedure as “perturbation with noise” can be confused with differential privacy mechanisms. I recommend “blinding” or “masking” for clarity.

*We replaced “perturbing” with “masking” throughout the manuscript for clarity.*

Minor comment #3: “In contrast to Cho et al., sPLINK is faster by a factor of -8 (1.25h vs. - 10.3h)”: Cho et al.’s method includes PCA. Is this a fair comparison? Also, I believe their Armitage trend test is equivalent to linear regression. Table 2 needs to be updated.

*We agree with the reviewer regarding the study by Cho et al., and therefore, we removed the corresponding comparison from the manuscript. We also updated Table2 and stated in the caption that “The study supports Cochran–Armitage test, which is computationally comparable to linear regression.”*

Minor comment #4: Figure 3a-c: I suggest explaining in the caption that the spike in noise in a specific region is due to strong association signals resulting in higher absolute error. Otherwise, these figures give the impression of statistical anomaly.

*We added this sentence to the caption of Figure3 to address that:*

*“The spikes in some genomic positions are due to the strong association of the corresponding*

*SNPs, which result in higher absolute error.”*

Minor comment #5: I disagree with the statement that HyFed is more secure than SMPC. In the event of collusion this is true, but when the respective security assumptions are held as intended, HyFed leaks poorly-understood amount of information through aggregated intermediate data whereas SMPC maintains security throughout the analysis.

*We agree with the reviewer that sPLINK is not better than SMPC from the privacy aspect. However, we think the claim that sPLINK provides weaker privacy compared to SMPC-based methods would be inaccurate too. This is because if the assumptions hold, only the aggregated parameters are revealed in sPLINK. In SMPC, the intermediate results from each computing party are shared with the other computing parties. For iterative algorithms, these intermediate results are revealed in each iteration, and therefore, we do not agree that “SMPC maintains security throughout the analysis”. As this aspect is debatable, we simply removed the privacy comparison between sPLINK and SMPC from the manuscript.*

**Third round of review**

**Reviewer 1**

The authors have more centrally featured the privacy considerations in the discussion. Issues remain in terms of the correctness and completeness of the presentation on this matter. See comments below.

The authors have not fully addressed the comment about the justification of HyFed compared to existing secure aggregation methods. The justification provided is that the prior work does not provide source code and includes additional extensions that are not needed, but this doesn’t explain the proposed techniques of HyFed which are very minimally described. Since it is a central component of sPLINK, its relation to existing works could be better described. One aspect that is especially lacking detail: the authors explain that the noise generated is random integer in range (-2^31, 2^31). This does not explain how floating-point values e.g. in the gradients are masked and aggregated in sPLINK. More mathematical details of this process are needed. Simply invoking the seminal work on secret sharing is not sufficient because the authors have not explained the transformation from the authors’ problems to the setting of secret sharing involving modular arithmetic.

The authors now classify all existing cryptographic solutions based on HE and SMPC as privacy-aware not privacy-preserving, thus putting them in the same category as federated learning in terms of privacy. This is not acceptable given the vastly more rigorous nature of HE/SMPC. The claim that there is no privacy guarantee with HE and SMPC is not accurate. I believe the discussion conflates privacy guarantee in the computation step vs. the leakage in the final model that is released. HE/SMPC does not address the latter but provides a guarantee for the former. This distinction needs to be further clarified.

The manuscript repeats the misleading statement in several places that SMPC shares “intermediate results” with other parties. This does not acknowledge that these intermediate results remain secret shares and thus do not leak any private information. Vast majority of works using SMPC only reveal the final results.

Differential privacy is a fundamentally different technique than HE/SMPC/FL that prevents leakage in the output (final or intermediate) as opposed to computation that leads to it (private disclosure vs. secure computation). The authors misleadingly portray it as the only “privacy-preserving” method.

The discussion throughout the manuscript emphasizes that sPLINK does not reveal local results among the servers however there is no clear information about what is being shared/revealed in sPLINK. Given the complexity of the proposed system, it is important that the readers get a clear picture of its requirements and assumptions. There needs to be a paragraph that clearly describes what constitutes the intermediate results/global parameters that are openly shared among the parties in sPLINK for different GWAS setups, and how this aspect compares with other approaches.

The manuscript misleadingly suggests that the possibility of reconstruction of private data from aggregated parameters in sPLINK is comparable to meta-analysis. Meta-analysis only shares association statistics whereas sPLINK shares higher dimensional data e.g. covariance matrix and therefore increases the risks. This point needs to be clarified.

Minor remark on Table 2 regarding completeness: The comment that “We systematically explored the robustness to heterogeneity only for tools offering chi-square, linear and logistic regression models” does not sound reasonable because whether a method implements different tests has nothing to do with robustness to heterogeneity. Since the authors’ main point here is that meta-analysis is sensitive to cross-dataset differences whereas any method that replicates centralized analysis is not, any of the other existing methods that are designed to replicate centralized analysis should get a checkmark.

Minor remark regarding “(IV) there are at least two different cohorts participating in the study. Under these assumptions, the original values of the local parameters of a single cohort remain hidden from the server, compensator, and the other cohorts”: If there are only two cohorts both cohorts can directly recover the local results of the other cohort by subtracting their own.

**Reviewer 3**

The proposed approach, though not perfect in its privacy guarantees, is an improvement relative to current approaches (e.g. meta-analysis). That said, there is a bigger concern (also articulated by the reviewer): the lack of details about the proposed HyFed scheme. This detail is also missing from the preprint on HyFed. While the theoretical properties of the secret sharing scheme are indeed well-known, there are non-trivial

issues in mapping GWAS and DGE analyses to the federated setting while being privacy aware. As one simple but important example, the secret sharing schemes such as the one that the authors refer to and rely on assume the data is finite e.g. integer-valued while the GWAS/DGE data are real-valued. Handling this gap is an important technical challenge that can potentially impact the privacy and/or the accuracy of the method. I could not find any clear description/discussion of this issue. In my mind, this is a serious gap in the submission.

**Authors Response**

**Point-by-point responses to the reviewers’ comments:**

Reviewer #3

Major comment : The proposed approach, though not perfect in its privacy guarantees, is an improvement relative to current approaches (e.g. meta-analysis). That said, there is a bigger concern (also articulated by the reviewer): the lack of details about the proposed HyFed scheme. This detail is also missing from the preprint on HyFed. While the theoretical properties of the secret sharing scheme are indeed well-known, there are non-trivial issues in mapping GWAS and DGE analyses to the federated setting while being privacy aware. As one simple but important example, the secret sharing schemes such as the one that the authors refer to and rely on assume the data is finite e.g. integer-valued while the GWAS/DGE data are real-valued. Handling this gap is an important technical challenge that can potentially impact the privacy and/or the accuracy of the method. I could not find any clear description/discussion of this issue. In my mind, this is a serious gap in the submission.

*We agree with the reviewer that these are important points to consider. We now describe the masking mechanism of sPLINK (inherited from HyFed) for both non-negative integers and floating-point numbers in the “Methods” section in detail:*

*“sPLINK inherits its masking mechanism from HyFed, which masks the local parameters with non-negative integer and floating-point values in different ways. For a local parameter with a non-negative integer value, sPLINK considers a finite field Zp ={0, 1, p − 1} (p is a prime number), where each client i generates a uniform random integer from Zp as noise N Li and masks its local parameter M Li with N Li by performing the modular addition over Zp : M′ Li =*

*(M Li + N Li ) mod p. Notice that M Li ,N Li ,M′ Li	Zp . For M Li with a floating-point value, each client i generates noise N Li using Gaussian random generator with zero-mean and variance 6 2 N , and masks M Li with N Li using the ordinary addition: M ′Li = M Li + N Li .*

*The compensator computes the aggregated noise N by taking sum over the noise values of the clients using the modular or ordinary addition depending on the data type of the noise: if N Li is non-negative integer, then N=(∑N Li ) mod p; if N Li is floating-point type, then N = ∑N Li . To calculate the global parameters with non-negative integer values, the server first computes the aggregated noisy parameter by taking sum over the noisy local parameters using the modular addition, and then subtracts the aggregated noise from the aggregated noisy parameter using the modular subtraction: M G = ((( ∑M′ Li ) mod p) - N) mod p. For model parameters with floating-point values, the server adds up the noisy local parameters and the negative of the aggregated noise using the ordinary addition: M G = ∑M ′ Li − N.”*

*We further discuss the impact of the masking mechanism parameters (the value of prime number p and variance of the Gaussian noise generator) on the correctness/precision of the results in the “Discussion” section:*

*“The value of prime number p impacts the correctness of the masking mechanism. To ensure the correctness, overflow must not occur in ∑N Li and ∑M′ Li calculations, and ∑M Li < p. sPLINK uses the default value of p = 2 54 − 33, which is the largest prime number than can fit in 54-bit integer. A higher value of p can be employed to handle larger integer values but at the expense of a lower number of clients. Likewise, too large values of variance 6 2 N (e.g. 10 30 ) can*

*impact the precision of the results. With default values of p and 6 2 N , however, our experiments indicate that there are no statistically significant differences between the results from sPLINK with and without the masking mechanism for all three association tests (The experimental setup of Figure 7 is used in the experiments).”*

*Finally, we now also discuss the privacy of the masking mechanism for both non-negative integers and floating-point values using the mutual information criterion as well as the effect of the masking mechanism parameters on privacy in the “Discussion” section:*

*“Given these assumptions, we discuss the privacy of the masking mechanism of sPLINK (inherited from HyFed) for the supported association tests. To this end, we use the information theoretic criterion called mutual information between two random variables X and Y :*

*I(X,Y ) = H(X) − H(X|Y ) where H(X) and H(X|Y ) indicate the entropy of X and the conditional entropy of X given Y , respectively. The mutual information measures (in bits) the decrease in uncertainty about X having the knowledge of Y . In sPLINK, the noisy local parameter M’ L is a secret share from the local parameter M L (the secret), and random variables X and Y indicate the distributions of M L and M’ L , respectively. The local parameter M L of a client is either a non-negative integer (e.g. sample count, allele count, or contingency table) or floating-point number (e.g. Hessian or covariance matrices) in the association tests.*

*For non-negative integers, sPLINK capitalizes on additive secret sharing based on modular arithmetic over the finite field Zp ={0, 1, p − 1}, in which p is a prime number . For floating-point numbers, sPLINK employs real value secret sharing based on Gaussian (Normal) distribution (more details in section 5).*

*For non-negative integers, noise N L is generated from a uniform distribution over Zp , and M′ L is the modular addition of M L and N L : M′ L = (M L + N L ) mod p. For this scheme, it has been shown that the knowledge of Y (noisy local parameter) provides no information about X (local parameter), which means the mutual information between them is zero: I(X,Y ) = 0. Notice that this is the case for any value of prime number p.*

*For floating-point numbers, noise N L is generated using Gaussian distribution with variance of σ 2 N . Assuming that the variance of X is σ 2 ML , the mutual information between X and Y is*

*maximum if Y follows the Gaussian distribution (variance σ 2 ML + σ 2 N ). Thus, the upper bound*

*on the mutual information between X and Y is:*

*I(X,Y ) = (1/2) log 2 (1 + σ 2 ML / σ 2 N )*

*That is, the amount of reduction in uncertainty about the local parameters having the knowledge of the noisy local parameters depends on the relative variance of the corresponding distributions. Therefore, using larger values for variance in the Gaussian random generator will provide lower information leakage. The value of mean for the Gaussian random generator does not remarkably impact the privacy and can be set to zero, which is the case for sPLINK. The default value of σ 2 N is 10 12 for sPLINK, which is large enough for typical GWAS, but it can be set to higher values if needed to ensure that σ 2 ML / σ 2 N remains small.”*

Reviewer #1

Minor comment #1: On Table 2 regarding completeness: The comment that “We systematically explored the robustness to heterogeneity only for tools offering chi-square, linear and logistic regression models” does not sound reasonable because whether a method implements different tests has nothing to do with robustness to heterogeneity. Since the authors’ main point here is that meta-analysis is sensitive to cross-dataset differences whereas any method that replicates centralized analysis is not, any of the other existing methods that are designed to replicate centralized analysis should get a checkmark.

*We updated Table2 and gave a checkmark to GLORE, and SMPC-based studies in the “Robust to heterogeneity” column.*

Minor comment #2: If there are only two cohorts both cohorts can directly recover the local results of the other cohort by subtracting their own.

*We agree with the reviewer on that, and thus, revised the assumption (IV) in the “Discussion” section: “there are at least three different cohorts participating in the study, and their client components as well as the server and compensator components are running in separate physical machines.”*

**Fourth round of review**

**Reviewer 2**

I am satisfied with the author reponses now. The claims of privacy are toned down and accurate now.
